# Reader-dependent functional duality of FTO: a context-switching node at the intersection of immune evasion and therapeutic resistance

**DOI:** 10.3389/fimmu.2026.1861781

**Published:** 2026-07-20

**Authors:** WenJie Tian, Bin Xie, Peng Zhan

**Affiliations:** 1 Department of Urology, the Second Hospital of Jilin University, Changchun, China; 2 Department of Biliary Surgery, Cancer Hospital of Dalian University of Technology, Cancer Hospital of China Medical University, Liaoning Cancer Hospital& Institute, Shenyang, Liaonin, China

**Keywords:** FTO, immune checkpoint regulation, metabolic reprogramming, therapeutic resistance, tumor immune microenvironment

## Abstract

The fat mass and obesity-associated protein (FTO), an RNA demethylase acting on both internal m^6^;A and cap-proximal m^6^;Am, functions in cancer as a context-dependent epitranscriptomic regulator whose net effect cannot be reduced to an oncogene–tumor-suppressor dichotomy. Its biological output is shaped by tumor lineage, subcellular localization, upstream signaling, and competing m^6^;A reader activities, predominantly YTHDF2-mediated decay and IGF2BP-mediated stabilization, although both reader families display additional non-canonical functions and are themselves modulated by post-translational modifications. Building on the now well-established context-dependence of FTO biology, which we do not claim as a novel observation, this review synthesizes current evidence on FTO’s roles at the intersection of tumor immune contexture, immune checkpoint regulation, metabolic reprogramming, and therapeutic resistance. We examine how FTO may contribute to immune exclusion through metabolic competition, exosomal signaling, and stromal reprogramming; modulate PD-L1 expression through direct and indirect mechanisms; and influence response to chemotherapy, targeted therapy, radiotherapy, and CNS-directed treatment. Emerging FTO inhibitors, FTO-degraders, and combination strategies with immune checkpoint blockade, ferroptosis inducers, or glycolytic inhibitors are evaluated against their underlying preclinical evidence base. The contribution of this review lies less in proposing a new framework than in three forms of integration typically addressed in isolation: explicit calibration of mechanistic claims to evidence tier, systematic separation of tumor-intrinsic from immune-cell-intrinsic FTO functions across lymphoid and myeloid compartments, and translation of reader-network biology into biomarker-stratified trial design. Technical limitations of epitranscriptomic methods are addressed as constraints on inference. To our knowledge, no FTO-targeted strategy has yet entered Phase I oncology evaluation; current combination rationales therefore remain preclinically supported rather than clinically established.

## 1 Introduction

Cancer remains a leading cause of mortality worldwide, and despite major advances in targeted therapies and immunotherapy, durable clinical responses are still limited by tumor heterogeneity, adaptive plasticity, and therapeutic resistance (1–4). Accordingly, the discovery of new therapeutic vulnerabilities and a deeper mechanistic understanding of tumor initiation and progression remain essential for advancing both early detection and effective treatment. In this context, RNA modifications have recently emerged as a major area of interest in cancer biology (5–7).

RNA modifications comprise a diverse set of chemical alterations affecting RNA nucleotides, with broad consequences for RNA conformation and function. To date, more than 170 distinct RNA modifications have been described across virtually all classes of RNA molecules (8). These epitranscriptomic marks regulate multiple aspects of RNA biology, including splicing, stability, subcellular localization, translation, and RNA–RNA as well as RNA–protein interactions, thereby shaping a wide range of cellular processes (9–11). Among the enzymes involved in this regulatory landscape, FTO was the first m6A demethylase to be identified and is now recognized as a key modulator of N6-methyladenosine (m6A) dynamics in mRNA (12). Beyond its function in m6A demethylation, FTO has also been implicated in regulating m6A and m6Am modifications in snRNA, as well as m1A demethylation in tRNA (13). Available evidence suggests that FTO preferentially acts on m6A sites within nuclear polyadenylated RNA, whereas in the cytoplasmic compartment it can also remove methyl groups from mRNA, with a notable preference for the cap-adjacent m6Am modification (13). As a reversible and highly dynamic epitranscriptomic mark, m6A has been implicated in fundamental biological processes, including the control of gene expression, RNA turnover, and cell fate decisions (14).

In the context of malignancy, FTO reshapes m6A patterns on selected transcripts, thereby contributing to tumor progression through the regulation of cellular aggressiveness, metastatic capacity, therapeutic resistance, and immune escape (15–21). FTO has emerged as a context-dependent epitranscriptomic regulator in cancer, influencing tumor progression, immune evasion, and therapeutic resistance in a manner that cannot be reduced to a simple oncogene–tumor suppressor dichotomy (22–25). Of particular relevance, its function is context-dependent: in hepatocellular carcinoma and bladder cancer, both oncogenic and tumor-suppressive roles have been reported (26–32), while in prostate and colorectal cancer FTO can either restrain or promote malignancy depending on the transcript context and downstream signaling output (33–37). These observations suggest that FTO functions as a context-switching regulatory node rather than a linear cancer driver (22–25). Mechanistically, the net biological consequence of FTO-mediated demethylation appears to depend on tumor lineage, target-transcript selection, and the balance of competing m6A readers, particularly YTHDF2- and IGF2BP2-dependent pathways (17, 38, 39). However, the field still lacks an integrated framework that explains and predicts why the same demethylase produces opposite biological outcomes across cancers. Moreover, most available evidence remains largely preclinical, underscoring the need for reader-network-based and lineage-aware models to guide translational targeting of FTO (17, 34, 35, 38, 39).

This review begins with a concise synthesis of FTO’s biological functions and its pivotal role in RNA epitranscriptomic regulation, before delineating the tumor-type-specific landscape of its activity and dissecting how it reshapes the tumor immune contexture through metabolic competition, immune checkpoint modulation, and remodeling of lymphoid and myeloid compartments. We subsequently integrate current evidence implicating FTO in resistance to chemotherapy, targeted therapy, radiotherapy, and immunotherapy, with a particular focus on the shared molecular nodes that mechanistically couple immune evasion to treatment failure. We then survey emerging pharmacological strategies against FTO and revisit the reader-dependent organizing logic that has emerged from recent literature, with particular attention to dimensions that remain underdeveloped in current syntheses: explicit evidence-tier calibration, the post-translational regulation of m^6^;A reader function itself, and the translation of reader-network biology into biomarker-stratified clinical trial design. By integrating these dimensions, this review aims to address an integration gap in the literature and to consider how context-aware, biomarker-guided targeting of FTO may contribute to more selective therapeutic approaches.

## 2 FTO in cancer: a concise biological primer

### 2.1 The FTO paradox and core molecular functions

A note on evidence calibration. The mechanistic evidence underlying current understanding of FTO in cancer is heterogeneous in its experimental rigor, and uncritical aggregation across evidence tiers can produce false-precision conclusions. Throughout this review, we have therefore attempted to calibrate every mechanistic claim to its underlying evidence base, distinguishing among five tiers: (i) mechanisms validated by genetic perturbation in multiple *in vivo* models with concordant patient-sample correlations; (ii) mechanisms supported by single *in vivo* perturbation studies (typically syngeneic or xenograft) with mechanistic supporting evidence; (iii) mechanisms demonstrated *in vitro* across multiple cell lines, awaiting *in vivo* confirmation; (iv) mechanisms reported in single cell-line or single-model experiments that establish a candidate axis but cannot, in isolation, support generalization; and (v) correlative associations derived from bulk transcriptomic, single-cell, or spatial profiling that identify candidate relationships but cannot establish causation. Where claims rest predominantly on tier (iv) or (v) evidence, we have explicitly indicated this limitation in the surrounding text rather than relegating it to a generic acknowledgment, and we have replaced framings such as “establishes,” “demonstrates,” and “orchestrates” with calibrated alternatives (“supports,” “indicates,” “is consistent with”) when the evidence does not justify the stronger claim. This approach is intended to preserve the conceptual integration that motivates a synthetic review while preventing the systematic upgrade of single-system findings into generalized biology, a pattern that has, in our view, frequently characterized the FTO literature itself.

FTO, identified as the first RNA demethylase shown to erase m6A in nuclear RNA (40), has since emerged as a regulator whose behavior in cancer does not conform to a simple oncogene-versus-tumor-suppressor dichotomy. Across the oncological landscape, FTO has been alternately implicated as a driver of leukemogenesis, melanoma progression and breast tumor aggressiveness, yet as a restraint on stem-like properties in colorectal carcinoma and on transformation in several other solid-tumor lineages (22–25). This “FTO paradox” is increasingly understood not as a literature-wide inconsistency but as the predictable output of a regulatory node whose net effect is dictated by converging contextual variables: the preferred substrate engaged (internal m^6^A versus cap-adjacent m^6^Am, often associated with cytoplasmic localization and displaying distinct transcript selectivity) (13, 25, 41), the subcellular compartment in which catalytic activity is executed, the lineage-specific transcriptomic landscape on which it operates, and the co-occurring activity of m^6^A writers and reader proteins that translate mark erasure into divergent downstream outputs (42). Collectively, emerging evidence suggests that this paradox likely reflects both biological context and methodological heterogeneity, whether studies interrogate bulk m^6^;A changes, cap-proximal m^6^;Am dynamics, or transcript-resolved stability and translation outputs, rather than a genuine biological ambiguity in FTO function. In contrast to the framing of FTO as a linear effector of m^6^A-dependent oncogenesis, a more coherent model positions it as a context-integrating node whose outputs are constrained by the chemical, spatial and cellular state in which it operates.

Layered onto this substrate- and compartment-level regulation is a post-translational architecture that tunes FTO activity in a context-dependent manner. Acetylation at conserved lysine residues — most notably K88, mediated by the acetyltransferase GCN5, enhances RNA engagement and demethylase activity without perturbing FTO stability, localization, or dimerization, and is elevated across several malignancies, providing a potential mechanistic link between nutrient and metabolic cues and epitranscriptomic rewiring (43). Ubiquitin-dependent turnover and incompletely mapped phosphorylation events further position FTO as a sensor of proteostatic and oxidative stress cues (44). A preprint study (awaiting peer review) additionally reports FTO localization to centrosomes, where it may support NuMA- and KIFC1-dependent spindle organization (45), if confirmed, this would extend FTO’s role beyond cap chemistry to the maintenance of mitotic fidelity in chromosomally unstable tumors. The integrated functional outputs of this node therefore span cancer-stem-cell reprogramming, metabolic rewiring of glycolytic and lipid-handling circuits, adaptive stress responses and genome-stability control, with the dominant output in any given tumor determined by which inputs prevail. Substantial knowledge gaps nonetheless persist: current methodologies do not always resolve the relative contributions of internal m6A and cap-adjacent m6Am, and they provide only limited spatial resolution of the nuclear and cytoplasmic pools through which FTO exerts substrate-selective effects (13). It is precisely this context dependence, rather than any unified pro- or anti-tumor identity, that predicts FTO’s divergent influence on the tumor-immune microenvironment and on sensitivity to immune-checkpoint blockade and targeted therapy (23, 46), a theme developed in the sections that follow.

### 2.2 The tumor-type landscape

Across the studies compiled in **Table 1**, encompassing gastrointestinal, hepatobiliary, genitourinary, breast and gynecological, lung, head-and-neck, central nervous system, pancreatic, hematological and other malignancies, FTO emerges not as a uniformly oncogenic or tumor-suppressive enzyme but as a context-dependent regulator whose net direction is dictated by tissue lineage and cellular state. A predominantly oncogenic profile has been reported in gastric, cervical, oesophageal squamous, ovarian, pancreatic ductal, nasopharyngeal and renal-cell carcinomas and in T-cell acute lymphoblastic leukemia, where elevated FTO supports proliferation, invasion, lymph-node dissemination and stem-like reprogramming (47–65). In contrast, FTO has been reported to function as a tumor suppressor in prostate carcinoma, papillary thyroid carcinoma, lung adenocarcinoma, glioma, multiple myeloma and osteosarcoma, and within the Epstein–Barr-virus-associated subtype of gastric cancer, where its loss accelerates growth, epithelial–mesenchymal transition and metastasis (66–76). Hepatocellular, bladder, breast and colorectal carcinomas occupy a third, internally inconsistent category in which both oncogenic and tumor-suppressive functions have been reported within the same tissue, sometimes with opposing prognostic correlations (26–32, 38, 39, 77–82). This distribution is unlikely to reflect experimental noise alone; rather, it may reflect the convergence of a shared catalytic activity onto distinct transcriptomic landscapes, reader-protein repertoires and upstream signals that sculpt FTO abundance and activity in each context.

**Table 1:** Cross-tumor landscape of FTO function in cancer.

Cancer type	FTO level	Role	Direct m6A/m6Am target(s)	Reader/mediator	Pathway/process	Functional output	Ref.
Colon cancer	↑	Onc	PKM2	—	Glycolysis (Warburg)	Proliferation, invasion, metastasis	(54)
CRC	↑	Onc	ZNF687	—	Wnt/β-catenin	Proliferation, invasion, migration, angiogenesis	(77)
CRC	↑	Regulatory	KCTD15 (anti-tumor)	YTHDF2	p53/HDAC1 axis	FTO stabilizes KCTD15 → ↓proliferation, ↑apoptosis	(34)
CRC	↓	TS (cohort)	HK2	IGF2BP2	FOXO/glycolysis (with ALKBH5)	↑Glycolysis, proliferation when FTO/ALKBH5 lost	(39)
CRC	↑ (USP30)	Onc	PHGDH, PSAT1	YTHDF2	Serine biosynthesis	USP30 stabilizes FTO → tumor growth	(82)
Gastric	↑	Onc	CCL2	YTHDF2 (antag.)	Autophagy ↓, ROS ↓	Proliferation, survival, tumor growth	(55)
Gastric	↑	Onc	MAP4K4	—	—	Proliferation, migration, invasion, ↓apoptosis	(56)
EBVaGC	↑ (MYC)	TS	FOS	IGF2BP1/2	—	Suppresses metastasis, invasiveness	(66)
Gastric	↑	Onc	GLG1	—	mRNA stability	Migration, invasion, metastasis	(173)
Gastric	↑	Onc	SP1, AURKB	—	ATM/p38 ↑, p53 ↓	Tumorigenesis, viability, colony, migration, invasion	(174)
Gastric (CagA+)	↑ (CagA→Jun)	Onc	HBEGF	—	EMT (hit-and-run)	Metastasis; persists after H. pylori eradication	(47)
Gastric	↑	Onc	circFAM192A → SLC7A5	—	Leucine uptake, mTOR	Proliferation	(48)
Gastric	↑	Onc	SOX2	—	Stemness	Tumor growth, stem-cell properties	(49)
Gastric	↑	Onc	FSP1	YTHDF2	Ferroptosis suppression	Proliferation, migration; AS-IV ↓FTO → ferroptosis	(85)
HCC	↑	Onc	BUB1	YTHDF2	TGF-β/TGF-βR1	Proliferation, invasion, EMT	(26)
HCC	↓	TS	VEGFA	—	Angiogenesis	FTO loss → growth, metastasis	(27)
HCC	↓ (nuclear)	TS	—	—	—	Reduced nuclear FTO → poor prognosis (clinical)	(28)
HCC	↓	TS	circGPR137B (feedback loop)	—	—	Suppresses tumorigenesis, invasion, lung mets	(29)
HCC	↑ (lncRNA FTO-IT1)	Onc	GLUT1, PKM2, c-Myc	YTHDF2 (antag.)	Glycolysis (c-Myc loop)	Proliferation, glycolysis	(53)
Bladder	↑	Onc	STAT3	—	STAT3 signalling	Proliferation, migration	(30)
Bladder	↓	TS	MALAT1, NOTCH1, CSNK2A2, ITGA6	SFPQ	5′/3′ UTR m6A modulation	↓Proliferation, ↓invasion, ↑apoptosis	(31)
Bladder	↓	TS	EMG1	—	Ribosome biogenesis	↓Proliferation, migration, invasion	(32)
Prostate	↓	TS	miR-139-5p (stab.) → ZNF217	—	PI3K/Akt/mTOR ↓	↓Proliferation, EMT, lung metastasis	(67)
Prostate	↓ in EMT	TS/EMT mod.	DDIT4	IGF2BP2/3	EMT	FTO loss → ↑motility, invasion, EMT	(33)
CRPC	↓ (DNA-damage Ub)	TS	FOXO3a	—	—	FTO loss → growth; FTO ↑ enhances PARPi response	(68)
Prostate	↑ (with ZFHX3 loss)	Onc context	E2F2, CDKN2C	—	Proliferation	ZFHX3 loss → ↑FTO activity → ↑growth	(37)
Prostate	↓	TS	CLIC4	—	mRNA stability	FTO loss → ↑proliferation, metastasis	(69)
Prostate	↓	TS	EGR2	—	Cell cycle, growth pathways	FTO ↑ → ↓proliferation, migration, invasion	(36)
ccRCC	↑	Onc	SLC1A5	—	Glutamine metabolism, reductive carbox., pyrimidines	↑Growth, survival; FTO inhibition → ↑ROS, DNA damage	(50)
Breast	↑	Onc	CYP27B1	HNRNPC	Vit-D ↓ → STAT3 ↑ (positive loop)	Tumor growth, chemoresistance	(78)
TNBC	↑	Onc	NFKBIE	—	NF-κB-related	Proliferation, migration, invasion	(79)
Breast	↑	Onc	lncRNA GAS5 (destab.)	IGF2BP2	QKI pathway	↑Proliferation (GAS5 normally tumor-suppressive)	(38)
Breast (HER2+)	↑	Onc	miR-181b-3p ↓ → ARL5B ↑	—	—	Migration, invasion	(80)
TNBC (metastatic)	Activated by uncouplers/2-HG ↓	TS (when active)	SHMT2, MTHFD2, MTHFD1L	—	Mitochondrial serine catabolism (MSCP)	FTO activation → ↓MSCP, ↓lung metastasis	(81)
Cervical	↑	Onc	FGF2	—	Lactate, growth	Proliferation, ↓apoptosis	(51)
Cervical	↑	Onc	ZEB1, Myc	—	EMT/proliferation	Proliferation, migration, invasion	(52)
Cervical	↑	Onc	PIK3R3	—	FoxO pathway	Proliferation, migration, invasion	(57)
Cervical	↑	Onc	BMP4	—	Hippo/YAP1/TAZ	Proliferation, colony, migration, invasion	(58)
Ovarian	↑	Onc	—	—	AKT phosphorylation, autophagy	Proliferation, ↓apoptosis, ↑autophagy	(59)
PTC	↓	TS	SLC7A11	—	Ferroptosis (m6A-independent for SLC7A11)	FTO ↑ → ferroptosis → ↓tumor	(70)
PTC	↓	TS	APOE	IGF2BP2	IL-6/JAK2/STAT3, glycolysis	FTO ↑ → ↓glycolysis, ↓growth	(71)
NSCLC	↑	Onc	FAP	YTHDF2	Integrin/FAK	Metastasis	(175)
LUAD	↓	TS	KDM5A (translation ↓)	YTHDF1	PTEN/PI3K/Akt	↓Proliferation, invasion, EMT	(72)
Oesophageal	↑ (METTL14 antag.)	Onc	AKT3	—	AKT signalling	Proliferation, tumorigenesis, metastasis	(60)
ESCC	↑	Onc	pri-miR-200b/a/429 (proc. ↓)	DGCR8 (impeded)	miR-200/EMT targets	Tumor growth, lymph-node dissemination	(61)
NPC	↑ (NOTCH1)	Onc	SPARC (in endothelium)	YTHDF2 (suppr.)	Tumor-endothelial crosstalk, vascular remodeling	Metastasis via exosomal FTO transfer	(64)
Glioma	↓	TS	EREG	—	PI3K/Akt → p53/p21 ↓	FTO ↑ → ↓proliferation, G1 arrest	(73)
GBM (T98G)	↓	TS	GSTO1	—	Oxidative stress, apoptosis	FTO ↑ → ↓proliferation, ↑apoptosis	(74)
Glioma TME (microglia)	Inhibited by R-2HG	TME modulator	IL-6	—	NF-κB pathway	R-2HG → ↓FTO → ↓microglial IL-6 production	(87)
PDAC	↑	Onc	C-Jun → PFKM	YTHDF2	Glycolysis	Tumorigenesis, metastasis	(62)
pNEN	↑	Onc	APOE → FASN	IGF2BP2	PI3K/AKT/mTOR, lipid metabolism	Tumor growth, lipid remodeling	(83)
Pancreatic	↑	Onc	—	—	CSC/EMT, p21/p27	Proliferation, EMT, stemness, tumorigenicity	(63)
PDAC	↑	Onc	ZEB1	—	TGF-β/EMT	Stemness, EMT (baicalein-targeted)	(84)
Pancreatic	↑	Onc	pri-miR-383-5p (proc. ↓)	IGF2BP1	ITGA3	Viability, metastasis, stemness	(86)
Pancreatic	↑	Onc	ADAMTS2, COL12A1, THBS2	—	ECM signalling	Migration, invasion	(176)
Multiple myeloma	↑	Onc	SERPINF1 (destab.)	IGF2BP1	Wnt/β-catenin, c-Myc, cyclin D1	Proliferation, tumor growth	(75)
T-ALL	↑	Onc	ELK3	—	Glycolysis (transcriptional)	Leukaemia initiation, progression	(65)
Osteosarcoma	↓ (TRIM17 Ub)	TS	PDK1	—	AKT/mTOR	FTO loss → ↑PDK1 → osteosarcoma malignancy	(76)

↑, increased expression, activation, or upregulation; ↓, decreased expression, inhibition, downregulation, or loss; →, mechanistic or regulatory relationship between molecular events.

Despite this directional heterogeneity, the downstream targets engaged by FTO converge on a strikingly small number of cancer hallmarks. Metabolic reprogramming is the most consistent output: a recurring theme across colorectal, hepatocellular, pancreatic and lymphoid malignancies is the demethylation of glycolytic enzymes and their transcriptional regulators, encompassing PKM2, GLUT1, HK2, PFKM and the ELK3-driven glycolytic program, to enhance Warburg-type flux (39, 53, 54, 62, 65), and this control extends to glutamine reprogramming, serine biosynthesis, mitochondrial serine catabolism and APOE-driven lipid metabolism in renal, colorectal, breast and pancreatic neuroendocrine tumors, respectively (50, 81–83). A second convergent axis links FTO to epithelial–mesenchymal transition and cancer-stem-cell self-renewal through demethylation of master regulators such as ZEB1 and SOX2 and components of NOTCH-, BMP- and Hippo/YAP-coupled signaling, sustaining invasion and tumor-initiating capacity in gastric, cervical, pancreatic and nasopharyngeal disease (49, 58, 63, 64, 84). In contrast, a third recurring theme positions FTO at the interface of stress adaptation, where it modulates oxidative balance, autophagic flux and ferroptotic vulnerability through transcripts that include SLC7A11, FSP1, GSTO1 and CCL2, with the direction of effect flipping between tumor types according to the prevailing redox state (55, 70, 74, 85). The signaling consequences of these target engagements ultimately funnel into a restricted set of core oncogenic axes, PI3K/AKT/mTOR, Wnt/β-catenin, TGF-β and JAK/STAT, whose activation or restraint is shared across nearly every tumour type considered (26, 67, 68, 72, 73, 75–78, 83). Distinct molecular substrates therefore converge on shared cancer hallmarks, supporting a model in which FTO functions less as a pathway-specific effector than as a regulatory hub that channels diverse epitranscriptomic perturbations into a constrained phenotypic output.

The contradictions within this landscape are most parsimoniously explained by a small number of intersecting variables. Across tumor types, lineage-specific dominance of particular m^6^A reader proteins most often YTHDF2 and IGF2BP1–3, redirects identical demethylation events towards opposite stability outcomes (33, 38, 66, 83, 86). differential reliance on m^6^A versus cap-adjacent m^6^Am substrates and on nuclear versus cytoplasmic FTO pools further partitions effects between transcript classes; and microenvironmental and oncogenic cues such as *Helicobacter pylori* CagA, EBV-encoded MYC activation, mitochondrial-uncoupling-derived 2-hydroxyglutarate and chronic inflammation can flip FTO’s net contribution within a single lineage (47, 66, 81, 87). A parallel layer of post-translational control by ubiquitin ligases and deubiquitinases gates FTO abundance in a metabolite-responsive manner, providing additional context-specific calibration (76, 82). Methodologically, the field remains over-reliant on cell-line and xenograft systems, on bulk MeRIP-seq without single-base or compartment-resolved mapping, and on limited clinical validation outside a few tumor types, leaving substrate assignments and direction-of-effect calls intrinsically vulnerable to revision. Collectively, the convergent regulation of metabolism, stemness, stress responses and core oncogenic signaling positions FTO as an upstream determinant of the tumor-cell phenotypes that most directly shape immune infiltration, cytokine output and antigen presentation (55, 64, 87), and that drive resistance to cytotoxic, targeted and immune-checkpoint therapies (68, 78) an interface examined in the sections that follow.

### 2.3 Germline variants and biomarker landscape

Convergent evidence positions FTO at the intersection of germline susceptibility, tumour biology and epitranscriptomic biomarker discovery. Germline variation exerts population- and lifestyle-dependent effects on cancer risk: the FTO rs9930506 polymorphism confers protection against colorectal cancer (CRC), particularly in non-smokers and non-drinkers, whereas neighboring variants such as rs9940128 show no comparable signal (88). At the tissue level, FTO is recurrently overexpressed and correlates with poor differentiation, nodal spread, advanced TNM stage and shortened survival in gastric cancer (89), a pattern echoed in pan-cancer analyses linking aberrant FTO expression to prognosis, tumor-infiltrating immune cell composition, immune checkpoint gene activity, mismatch-repair status, microsatellite instability and tumor mutational burden across diverse malignancies (90). Complementarily, circulating m6A regulators and downstream targets such as SOX2 achieve high discriminatory accuracy for early CRC detection and staging, supporting an epitranscriptomic liquid-biopsy paradigm (91). Yet methodological heterogeneity, divergent assays (PCR-RFLP, IHC, qPCR, in silico mining), inconsistent thresholds, modest cohorts, and scarce prospective validation, together with still-superficial integration with established immune biomarkers, constrains clinical translation. Embedding standardized, multilayered FTO assessment, across germline, tumor, and circulating compartments, within immune-molecular panels has been proposed as a route to more refined patient stratification, but this proposition is currently hypothesis-generating rather than clinically validated. Establishing whether FTO-related biomarkers add independent predictive value beyond established ICI-response markers (PD-L1 IHC, TMB, MSI, gene-expression signatures) will require prospective testing in adequately powered, treatment-defined cohorts. We highlight this as a research priority rather than a present-day clinical recommendation.

## 3 FTO as an architect of the tumor immune contexture

Before surveying FTO’s roles within the tumor immune contexture, it is important to clarify a distinction that runs through the entirety of this section and carries direct therapeutic consequences: FTO operates within two functionally distinct cellular compartments, tumor-cell-intrinsic and immune-cell-intrinsic, and its effects in each compartment are not only different but in several cases directionally opposed. Tumor-cell-intrinsic FTO is implicated in metabolic competition, checkpoint-ligand stabilization, exosomal cargo regulation, and stromal reprogramming, and its inhibition is generally associated with restored anti-tumor immunity. Immune-cell-intrinsic FTO, by contrast, can support effector-cell function: in CD8^+^ T cells it sustains survival by limiting Fas-driven apoptosis (92), and recent work indicates it may also be required for productive CD4^+^ Th1 differentiation in non-cancer settings; in NK cells, however, FTO restrains cytotoxic activation (46), illustrating that the immune-cell-intrinsic effect is itself lineage-specific. The therapeutic implication is consequential: a non-selective systemic FTO inhibitor would simultaneously act on both compartments, and the net outcome, anti-tumor immune restoration versus impairment of CD8^+^ persistence, cannot be predicted from tumor-cell data alone. Throughout the sections that follow, we have endeavored to specify whether each cited mechanism describes FTO activity within the tumor compartment, within a defined immune-cell lineage, or, in the case of bulk-tissue correlative studies, whether the cellular source of FTO activity is not resolvable.

### 3.1 Tumor-intrinsic immune rewiring through metabolic competition

Across multiple solid tumors and hematological malignancies, FTO operating within the tumor cell itself (i.e., tumor-cell-intrinsic FTO) has been implicated in metabolic reprogramming, secretome remodeling, and checkpoint-ligand stabilization that together contribute to an immunosuppressive microenvironment and impaired CD8^+^ T-cell function (**Table 2**). The immune effects discussed in this subsection are downstream consequences of tumor-cell FTO activity rather than evidence of FTO activity within infiltrating immune cells. Initial mechanistic evidence for this axis was provided by a study demonstrating that FTO elevates c-Jun, JunB, and C/EBPβ to reprogram glycolytic flux, directly depleting nutrients available to infiltrating CD8^+^ T cells and impairing their effector function (93). This metabolic competition model is extended in HCC, where FTO stabilizes FLAD1 through suppression of YTHDF2-mediated mRNA degradation, coupling m^6^;A-dependent metabolic regulation to PD-L1 upregulation and reduced T-cell cytotoxicity (17). Notably, a parallel HCC study revealed a non-metabolic but convergent arm of the same axis: FTO-stabilized GPNMB is packaged into small extracellular vesicles and delivered to CD8^+^ T cells via SDC4, imposing paracrine inhibitory signals that reinforce immune exclusion independently of nutrient competition (94). In gastric cancer, FTO expression correlates with immunosuppressive TME scores, immune checkpoint gene upregulation, and m^6^;A-dependent TGF-β regulation, suggesting that the axis operates through cytokine-mediated tolerance beyond direct metabolic effects (95). In leukemia, HHT-induced FTO degradation suppresses LILRB4 to restore CD8^+^ T-cell cytotoxicity (96), while pharmacological FTO inhibition simultaneously attenuates cancer stem cell self-renewal and downregulates immune checkpoint programs including LILRB4 (97), findings that collectively highlight a shared vulnerability across disease contexts.

**Table 2:** Role of FTO in tumor immune evasion and antitumor immunity: mechanistic, pharmacologic, and correlative evidence with cross-study synthesis.

Cancer/model	FTO status/expression	Key downstream axis/target	Immune effect (Principal Finding)	Cellular compartment	Evidence tier	Mechanistic category	Therapeutic implication	Ref.
Multiple solid tumors; syngeneic mouse models	Upregulated; tumor-intrinsic	c-Jun, JunB, C/EBPβ; glycolytic reprogramming	CD8^+^ T cells: functional suppression via FTO-driven glucose depletion in the TME	Tumor-intrinsic	ii	Metabolic–immune	Dac51 (FTO inhibitor) + anti-PD-1 synergy	(93)
HCC; *in vitro*/*in vivo*; TCGA bioinformatics	Upregulated; tumor-intrinsic	FTO–YTHDF2–FLAD1 axis; PD-L1	CD8^+^ T cells: PD-L1 upregulation suppresses cytotoxicity (↓ IFN-γ, TNF-α, LDH release)	Tumor-intrinsic	ii	Metabolic + Checkpoint	FTO–YTHDF2–FLAD1 axis as combinatorial therapeutic target	(17)
HCC; *in vitro*/*in vivo*	Upregulated; tumor-intrinsic	FTO/m^6^;A/GPNMB; sEV-GPNMB–SDC4	CD8^+^ T cells: sEV-GPNMB inhibits activation via SDC4 binding; reduced TIL recruitment	Tumor-intrinsic	ii	Exosomal signaling	CS2 (FTO inhibitor) sensitizes to anti-PD-1 and sorafenib	(94)
Acute monocytic leukemia; THP-1 cells, AML xenograft, patient samples	Pharmacologically degraded by HHT	FTO/m^6^;A/MLL1/LILRB4	CD8^+^ T cells: LILRB4 downregulation → enhanced cytotoxicity and immune activation	Tumor-intrinsic (paracrine effect on CD8^+^)	iii	Checkpoint (non-PD-L1)	HHT (FDA-approved for CML) repositioned for AML immunosensitization	(96)
AML; *in vitro*/*in vivo*	Genetically depleted; pharmacologically inhibited (novel small-molecule inhibitors)	LILRB4; immune checkpoint genes; CSC self-renewal	CD8^+^ T cells: checkpoint gene suppression; restored cytotoxicity; reversed hypomethylating agent-induced immune evasion	Tumor-intrinsic	iii	Checkpoint + Stemness	FTO inhibitors as dual-target agents (CSC + immune evasion)	(97)
Melanoma; syngeneic mouse models	Pharmacologically degraded by VES via DTX2/UFD1 ubiquitin-proteasome axis	DTX2–UFD1/FTO ubiquitination; FTO/m^6^;A/LIF	T cells: reduced LIF expression sensitizes tumor cells to cytotoxicity; overcame immunotherapy resistance	Tumor-intrinsic	ii	Cytokine modulation	VES (dietary FTO degrader) + anti-PD-1; DTX2/UFD1 as actionable regulatory axis	(100)
CD8^+^ T-cell immunity; T cell-specific Fto knockout mice; tumor-relevant context	T cell-specific genetic deletion	FTO/m^6^;A/Fas–IGF2BP3	CD8^+^ T cells: FTO loss → IGF2BP3-mediated Fas mRNA stabilization → apoptosis → impaired effector response and antitumor immunity	Immune-cell-intrinsic (CD8^+^)	ii	Apoptosis (Fas axis)	FTO supports CD8^+^ T-cell survival; cell-compartment specificity essential for therapeutic design	(92)
Melanoma (*in vivo*); leukemia (*in vitro*); Fto−/− mouse and human NK cells	Genetically depleted (Fto−/−)	FTO/m^6^;A/SOCS family; IL-2/IL-15–JAK/STAT signaling	NK cells: FTO depletion → reduced SOCS mRNA stability → JAK/STAT hyperactivation → NK hyperactivation and enhanced cytotoxicity	Immune-cell-intrinsic (NK)	ii	JAK-STAT regulation	FTO inhibition in NK cells as allogeneic NK-cell immunotherapy strategy	(46)
Thyroid carcinoma; xenograft + THP-1-differentiated macrophages	Upregulation (tumor-suppressive context)	FTO/m^6^;A/RASGRF1 (m^6^;A-dependent mRNA destabilization)	M2 macrophages: FTO-mediated RASGRF1 mRNA destabilization → reduced M2 polarization and migration	Tumor-intrinsic (acting on secretome)	iii	Myeloid (M2 reduction)	Anti-RASGRF1 strategies; FTO as tumor-suppressive axis in THCA	(103)
ESCC; *in vitro*	Elevated; degraded via FOXF2/RNF144A-mediated ubiquitination	FOXF2 → RNF144A (E3 ubiquitin ligase) → FTO ubiquitination/degradation	M2 TAMs: FTO degradation → increased m^6^;A → reduced M2 polarization and immunosuppressive TME	Tumor-intrinsic	iii	Myeloid (M2 reduction)	FOXF2/RNF144A/FTO axis as therapeutic target in ESCC	(102)
NSCLC; *in vitro* + xenograft	Upregulated; knockdown tumor-suppressive	FTO/m^6^;A/KCNAB2; PI3K/AKT pathway	M2 macrophages: FTO knockdown restores KCNAB2 → PI3K/AKT inactivation → reduced M2 polarization	Tumor-intrinsic (acting on secretome)	iii	Myeloid (M2 reduction)	FTO–KCNAB2–PI3K/AKT axis as NSCLC therapeutic target	(18)
LUAD; *in vitro* + nude mouse xenograft	Upregulated; positively correlated with QPCT expression	FTO/m^6^;A/QPCT–CCL2	M2 macrophages (CD68^+^/CD206^+^): stabilized QPCT pyroglutamylates CCL2 → macrophage chemotaxis and M2 polarization	Tumor-intrinsic (acting on secretome)	iii	Myeloid (M2 promotion)	FTO/QPCT/CCL2 axis; QPCT inhibition as candidate strategy	(101)
CRC; AOM/DSS mouse model + *in vitro*	Overexpression (tumor-suppressive context)	FTO/m^6^;A/CSF3–RLN2; NETosis	Neutrophils: FTO demethylation of CSF3 mRNA → reduced CSF3/RLN2 → suppressed NETosis	Tumor-intrinsic (acting on secretome)	iii	Myeloid (NETosis suppression)	m^6^;A–CSF3–RLN2 pathway as CRC therapeutic target	(104)
Gastric cancer; TCGA-STAD bioinformatics + limited functional validation (IHC, WB, qPCR)	Upregulated; associated with worse prognosis (correlative)	FTO/m^6^;A/TGF-β; PI3K/AKT, cAMP signaling (bioinformatic association)	Multiple immune cell types: FTO positively correlated with immune infiltration, checkpoint gene expression, and elevated TME score (associative)	Tumor-intrinsic (correlative)	v	Cytokine (correlative)	FTO as independent prognostic biomarker in GC	(95)
Gastric cancer; scRNA-seq, spatial transcriptomics, bulk cohorts (5 datasets)	Elevated in immune-excluded phenotype; correlated with stromal activation (associative)	FMR1–FTO (post-translational stabilization); CAF-collagen-integrin; MIF signaling	CD8^+^ T cells excluded from tumor core by CAF-collagen barrier; FMR1 stabilizes FTO protein; spatial/associative evidence	Tumor-intrinsic + Stromal	v	Spatial/Stromal exclusion	FMR1–FTO axis as candidate target to reverse immune exclusion and improve ICI sensitivity	(105)
Gastric cancer; TCGA + GTEx bioinformatic analysis	Upregulated; component of m^6^;A prognostic signature (bioinformatic)	m^6^;A regulator landscape (FTO, METTL3, METTL14, et al.); PD-L1/PD-1	Multiple immune subsets (NK, Treg, CD4^+^): FTO correlated with PD-L1, PD-1, and TIME; CNV affects immune infiltration (bioinformatic only)	Tumor-intrinsic (correlative)	v	Multi-axis (correlative)	m^6^;A regulator profiles as independent prognostic risk scoring in GC	(98)

Cellular compartment indicates the cell type in which FTO is acting: *Tumor-intrinsic*, FTO functions within tumor cells; *Immune-cell-intrinsic*, FTO functions within the named immune-cell lineage; *Stromal*, fibroblast/vascular compartment involvement.

Evidence tier (per the Evidence Calibration framework introduced in Section 2.1): (i) mechanisms validated by genetic perturbation in multiple *in vivo* models with patient-sample correlation; (ii) single *in vivo* perturbation study with mechanistic support; (iii) multi-cell-line *in vitro* evidence; **(iv)** single-cell-line or single-model evidence; **(v)** correlative bioinformatic or transcriptomic evidence only.

Mechanistic category groups studies by primary mechanism to facilitate cross-study pattern recognition.

Pattern note. Across **Table 2**, the dominant pattern is that tumor-cell-intrinsic FTO activity converges on CD8^+^ T-cell suppression through three non-mutually-exclusive mechanisms: glycolytic competition, metabolic–checkpoint coupling, and exosomal signaling. Findings from immune-cell-intrinsic FTO perturbation are directionally opposed to tumor-intrinsic findings and underscore the need for compartment-selective therapeutic strategies. Evidence tier distribution: the strongest evidence (Tier ii) supports the pan-tumor glycolytic, hepatocellular metabolic–checkpoint, and CD8^+^/NK lineage-specific mechanisms; correlative bioinformatic evidence (Tier v) dominates the gastric cancer literature on this axis.↓ indicates decreased expression, activity, abundance, or immune function; → indicates a mechanistic or regulatory pathway linking FTO to downstream immune or molecular events.

Despite these convergent findings, critical limitations temper interpretation. The depth of metabolic investigation varies substantially: while glycolytic competition is mechanistically dissected in (93) and FLAD1/FAD metabolism in (17), whereas other studies (94–97) offer limited direct evidence of nutrient competition as the primary driver, raising the question of whether metabolic rewiring is hierarchically upstream of the exosomal and checkpoint mechanisms or merely co-occurring. Furthermore, most evidence derives from knockdown or pharmacological inhibition models rather than endogenous FTO dysregulation in primary human tumors, and single-cell or multi-omics resolution of the TME remains largely absent. The cancer-type specificity of individual mechanisms particularly the HCC-restricted exosomal axis (94) and AML-specific LILRB4 regulation (96, 97) limits generalizability across tumor contexts.

**Figure 1:**
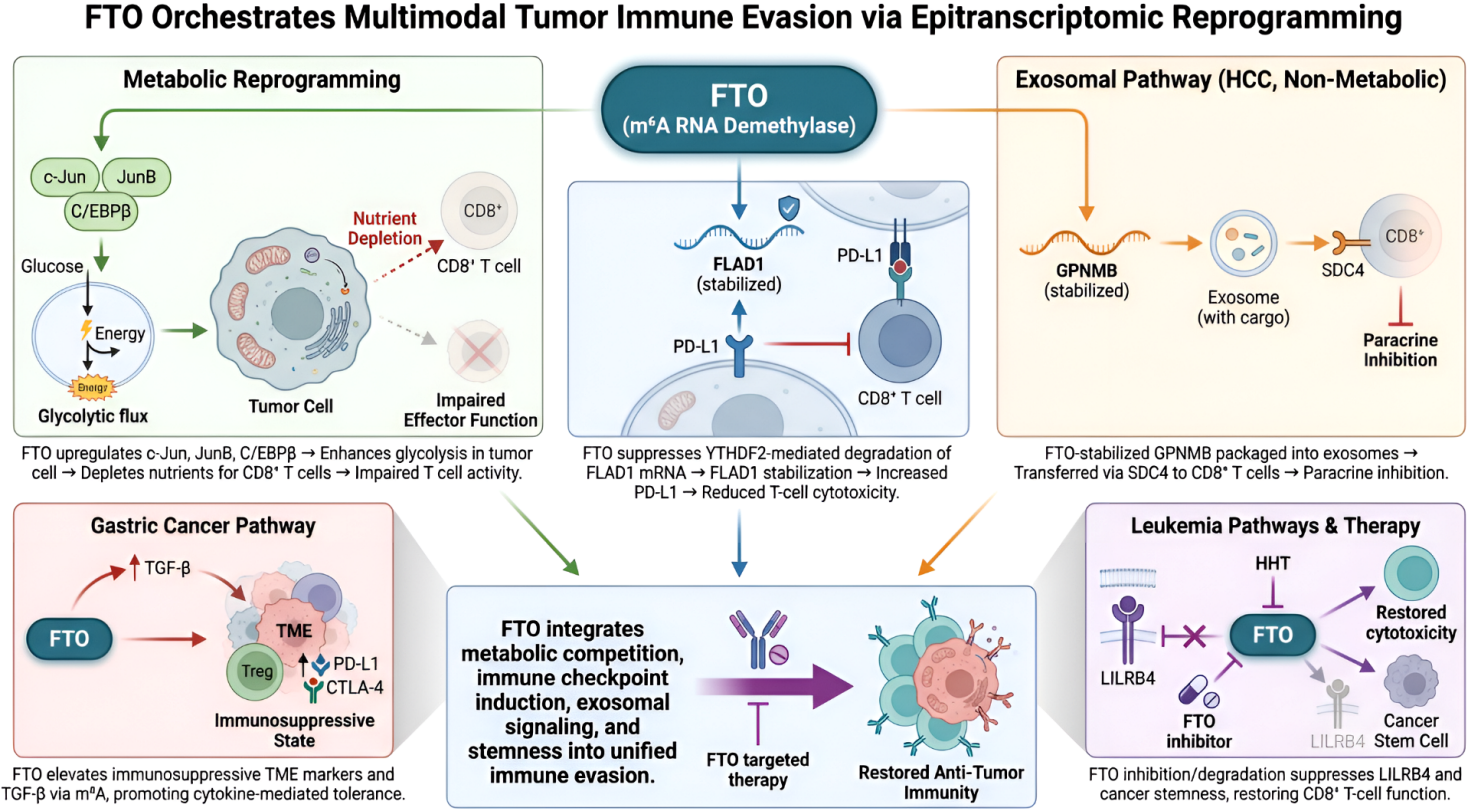
Tumor-cell-intrinsic mechanisms by which FTO has been linked to CD8^+^ T-cell suppression across cancer types. Pathways shown include glycolytic reprogramming (c-Jun/JunB/C/EBPβ; FLAD1), checkpoint induction (PD-L1), exosomal signaling (GPNMB–SDC4), cytokine modulation (TGF-β), and leukemia-specific checkpoint regulation (LILRB4). See main text and **Table 2** for evidence tiers and references.

Collectively, these studies indicate that FTO contributes to multiple immune evasion mechanisms, metabolic competition, checkpoint induction, exosomal signaling, and stem-cell maintenance, across cancer contexts. Pharmacological FTO targeting, via purpose-built inhibitors or repurposed agents such as HHT, has been proposed as a candidate strategy for simultaneous disruption of these layers (**Figure 1**).

### 3.2 Lymphoid compartment: CD8^+^ T cells and NK-cell cytotoxicity

The studies discussed in this subsection address FTO activity within lymphoid cells themselves (i.e., immune-cell-intrinsic FTO), in contrast to the preceding subsection on tumor-cell-intrinsic FTO function. Within the lymphoid compartment, FTO performs context-dependent and, in some settings, opposing functions across cell lineages; clarifying these lineage-resolved roles is necessary to understand how FTO shapes anti-tumor cytotoxicity at the systems level and to anticipate the consequences of pharmacological FTO inhibition on immune-cell function (**Table 2**).

In CD8^+^ T cells, where FTO is expressed cell-intrinsically and acts on lymphocyte transcripts directly, FTO functions as a pro-survival regulator through control of Fas-mediated apoptosis. T cell-specific FTO deletion produces a quantifiable survival defect: in the LM-OVA co-transfer model, FTO-deficient CD8^+^ T cells showed an approximately twofold increase in Annexin V^+^ apoptotic frequency at day 5 after infection, accompanied by an approximately 60% reduction in splenic donor CD8^+^ T-cell recovery relative to wild-type controls. Mechanistically, FTO ablation increased m^6^;A methylation of Fas mRNA, enhanced Fas mRNA stability, and elevated Fas surface expression by approximately 1.2–1.7-fold depending on the activation setting and time point. This effect was linked to IGF2BP3-dependent reader activity, as mutating the major Fas m^6^;A sites or knocking down IGF2BP3 attenuated Fas upregulation and the associated apoptotic phenotype. These data support a direct FTO–m^6^;A–IGF2BP3–Fas axis controlling CD8^+^ T-cell survival rather than a merely correlative association (92). These data indicate that FTO supports T-cell persistence in this context rather than functioning as an immunosuppressor, in contrast to its tumor-cell-intrinsic role in dampening anti-tumor immunity. Whether FTO performs analogous functions in CD4^+^ T helper cells within the tumor microenvironment remains an unresolved question, and the available evidence is markedly thinner than for CD8^+^ or NK lineages. No study, to our knowledge, has yet performed lineage-specific FTO perturbation in tumor-infiltrating CD4^+^ T cells, and the field therefore lacks direct mechanistic evidence linking CD4^+^-intrinsic FTO activity to Th1, Th17, Tfh, or Treg programming in any cancer context. The only currently available cancer-context data are correlative: in gastric cancer, m^6^;A regulator–based bioinformatic analyses associate FTO expression with broader immune infiltrate composition including CD4^+^ subsets, but these signals derive from bulk transcriptomes and cannot distinguish tumor-cell-intrinsic from CD4^+^-intrinsic FTO biology (98). Outside cancer, T cell–specific Fto deletion in a pathogen-challenge model has been reported to constrain Th1 effector expansion, T-bet expression, and IFN-γ production (99), a finding that is mechanistically suggestive given the central role of Th1 help and IFN-γ in productive anti-tumor responses and ICI efficacy, but which has not been validated in a tumor setting and therefore cannot be assumed to translate. This gap is consequential: CD4^+^ T-helper subsets shape CD8^+^ priming, sustain memory responses, license dendritic cells, and directly produce IFN-γ within tumors, and the polarity of FTO-dependent regulation across Th1/Th17/Treg axes in the TME is currently unknown. Resolving this question will require lineage-restricted Fto deletion (e.g., Th1-, Treg-, or Tfh-specific Cre drivers), CD4^+^ TIL–focused single-cell m^6^;A profiling, and adoptive transfer experiments in syngeneic tumor models. Until such evidence is generated, claims that pharmacological FTO inhibition will not perturb CD4^+^ help should be treated as untested, and this consideration further reinforces the broader argument for cell-compartment-selective rather than systemic FTO targeting.

FTO-deficient NK cells exhibit a quantifiable gain-of-function phenotype. In the B16F10 melanoma lung-metastasis model, Fto⁻/⁻ mice showed an approximately 60% reduction in macroscopic pulmonary metastatic nodules relative to wild-type controls, and NK-cell depletion largely abolished this protection, indicating that the phenotype was NK-cell dependent (46). Consistently, FTO knockdown in human NK92 cells enhanced cytotoxicity against K562 leukemia targets by approximately 1.6-fold at standard effector-to-target ratios. Mechanistically, FTO negatively regulates IL-2/IL-15-driven JAK/STAT signaling by sustaining the expression of SOCS-family negative-feedback regulators, including SOCS1 and SOCS3 (46). Loss of FTO increases m^6^;A methylation on SOCS transcripts, reduces SOCS mRNA/protein abundance, and thereby releases JAK/STAT signaling from negative-feedback constraint, resulting in enhanced NK-cell cytotoxicity, cytokine production, and antitumor activity (46). This inverse relationship, FTO supporting CD8^+^ survival while restraining NK cytotoxicity, is consistent with a cell-type-specific regulatory logic rather than a uniformly immunosuppressive function.

VES-induced FTO degradation, mediated through enhanced FTO–DTX2 interaction and UFD1-dependent recognition of ubiquitinated FTO, sensitized B16F10 melanoma tumors to anti-PD-1 therapy, with VES plus anti-PD-1 reducing endpoint tumor weight by approximately 65–70% relative to anti-PD-1 alone. (100). Rather than reducing LIF expression, VES or genetic FTO inhibition increased m^6^;A enrichment on LIF transcripts and decreased LIF mRNA decay, thereby increasing LIF mRNA stability. LIF overexpression enhanced T-cell-mediated melanoma-cell killing, while FTO overexpression counteracted this effect, supporting LIF as an important downstream mediator of tumor-intrinsic FTO-dependent immune resistance (100).

Critical limitations persist across this body of work. The apparent contradiction between FTO’s pro-survival role in CD8^+^ T cells (92) and its immunosuppressive role in tumors (100) remains mechanistically unresolved and underscores the danger of generalizing FTO inhibition as uniformly immunostimulatory. These studies rely on genetic knockouts or exogenous compounds rather than endogenous regulatory perturbations in primary human tumors, and single-cell resolution of lymphoid heterogeneity is entirely absent. Whether FTO inhibition selectively harms CD8^+^ T-cell persistence while simultaneously unleashing NK cytotoxicity represents an unaddressed therapeutic trade-off that demands lineage-resolved *in vivo* modeling.

**Figure 2:**
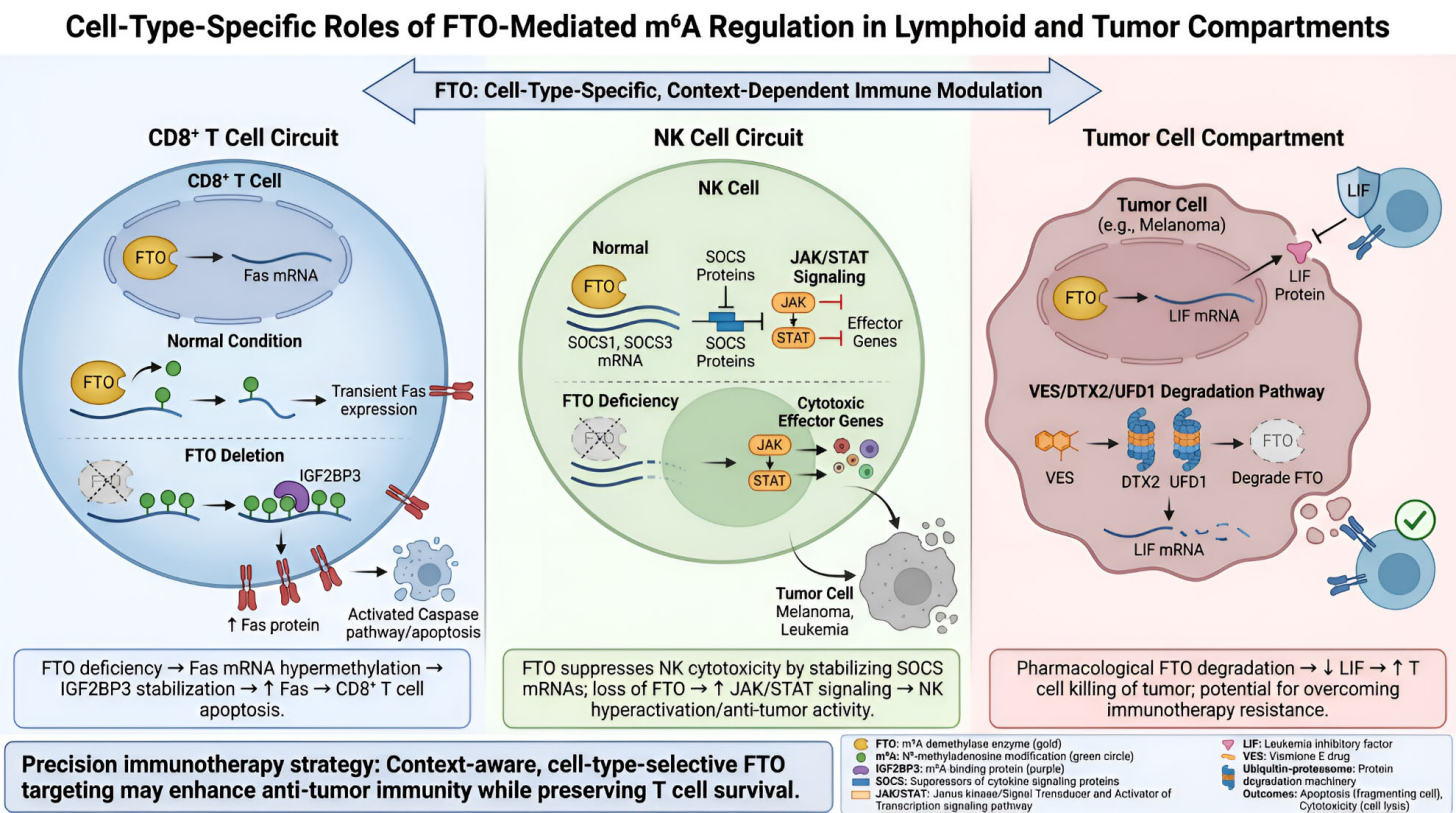
Lineage-specific FTO functions within the lymphoid compartment. FTO supports CD8^+^ T-cell survival by limiting IGF2BP3-mediated stabilization of Fas mRNA (left) and restrains NK-cell cytotoxicity by stabilizing SOCS transcripts that attenuate JAK/STAT signaling (right). Tumor-cell-intrinsic FTO-mediated LIF expression and its pharmacological degradation (VES–DTX2/UFD1 pathway) are also depicted. See main text for references.

The translational implication of this lineage-resolved analysis is consequential: a non-selective systemic FTO inhibitor would simultaneously act on tumor cells (anti-tumor effect), CD8^+^ T cells (potentially detrimental, via loss of FTO-mediated Fas-apoptosis restraint), CD4^+^ T cells (uncertain effect, given the limited tumor-context data), and NK cells (potentially beneficial, via release from FTO-mediated SOCS stabilization). The net immune-compartment outcome therefore cannot be predicted from preclinical tumor-cell data alone. Tumor-selective or compartment-restricted FTO targeting, through tumor-cell-directed delivery, conditional protein-degrader strategies, or biomarker-driven patient stratification, should be considered a prerequisite for clinical development rather than an optional refinement (**Figure 2**).

### 3.3 Myeloid compartment: macrophage polarization and NETosis

A particular feature of FTO biology in the myeloid compartment merits emphasis at the outset: with currently available evidence, the dominant FTO activity shaping macrophage polarization and neutrophil function in tumor settings is tumor-cell-intrinsic rather than macrophage-intrinsic. That is, FTO within tumor cells regulates secreted signals (chemokines, cytokines, exosomal cargo) that, in turn, polarize tumor-infiltrating myeloid cells. To our knowledge, no published study has performed myeloid-lineage-specific FTO deletion in a tumor context, and the cell-autonomous role of FTO within tumor-infiltrating macrophages or neutrophils therefore remains unresolved. The mechanisms described in this subsection should be interpreted accordingly. Across thyroid cancer, esophageal squamous cell carcinoma, non-small cell lung cancer, lung adenocarcinoma, and colorectal cancer, FTO-mediated m^6^;A demethylation emerges as a recurrent regulator of myeloid behavior yet its directional effects are strikingly context-dependent, oscillating between pro-tumorigenic immunosuppression and tumor-restraining myeloid reprogramming (**Table 2**).

The pro-immunosuppressive arm of FTO activity is most clearly articulated through its control of tumor-secreted myeloid-modulating signals. In lung adenocarcinoma, FTO-mediated demethylation stabilizes QPCT mRNA, enabling QPCT to modify CCL2 into a pyroglutamylated, stable form that drives macrophage chemotaxis and M2 polarization (101). Similarly, in NSCLC, FTO suppresses m^6^;A methylation of KCNAB2, maintaining its low expression and thereby sustaining PI3K/AKT signaling to license M2 macrophage polarization (18). Critically, both mechanisms operate through tumor cell-secreted or tumor cell-regulated intermediaries rather than through direct FTO activity within macrophages a distinction that fundamentally repositions FTO as a regulator of the tumor secretome rather than a macrophage-intrinsic epigenetic programmer. This distinction is reinforced by the ESCC context, where the FOXF2–RNF144A axis promotes FTO ubiquitination and proteasomal degradation in tumor cells, and the consequent FTO loss reduces TAM M2 polarization (102), again implicating tumor-intrinsic FTO as the operative node.

Paradoxically, in thyroid cancer, FTO upregulation destabilizes RASGRF1 mRNA via m^6^;A-dependent mechanisms, and this destabilization suppresses rather than promotes both tumor progression and macrophage M2 polarization (103). This apparent contradiction reveals that FTO’s downstream myeloid consequences are dictated entirely by target gene identity: when FTO stabilizes oncogenic mediators such as QPCT, it promotes immunosuppression; when it destabilizes oncogenic drivers such as RASGRF1, it restrains it. FTO is therefore not an intrinsically immunosuppressive enzyme but a context-dependent epitranscriptomic switch whose polarity is determined by the m^6^;A landscape of individual tumor types.

Beyond macrophages, FTO shapes the neutrophil compartment through a discrete regulatory axis. In colorectal cancer, FTO-mediated demethylation of CSF3 mRNA suppresses CSF3 expression, which in turn reduces RLN2 levels and attenuates NETosis a form of neutrophil cell death that paradoxically promotes tumor progression (104). FTO overexpression in this context reduces tumor burden and disease severity *in vivo*, positioning FTO as a NETosis suppressor and, counter-intuitively, as a tumor-restraining factor in the neutrophil compartment. This is mechanistically distinct from its macrophage-regulatory roles and underscores the cell-type specificity of FTO’s myeloid effects.

Several critical limitations constrain interpretive confidence across this body of evidence. All macrophage polarization studies rely on conditioned medium or indirect co-culture systems rather than primary human tumor-associated macrophages, rendering the physiological relevance of observed polarization shifts uncertain. Single-cell or spatially resolved analysis of myeloid infiltrates is entirely absent, precluding assessment of whether FTO-regulated signals reshape defined TAM subpopulations or broadly alter myeloid composition. Furthermore, the cross-cancer heterogeneity of FTO’s effects tumor-suppressive in thyroid and colorectal cancers, tumor-promoting in lung substantially limits therapeutic generalizability and raises the unresolved question of whether tissue-specific m^6^;A target hierarchies, rather than FTO expression per se, determine net immunological outcome.

**Figure 3:**
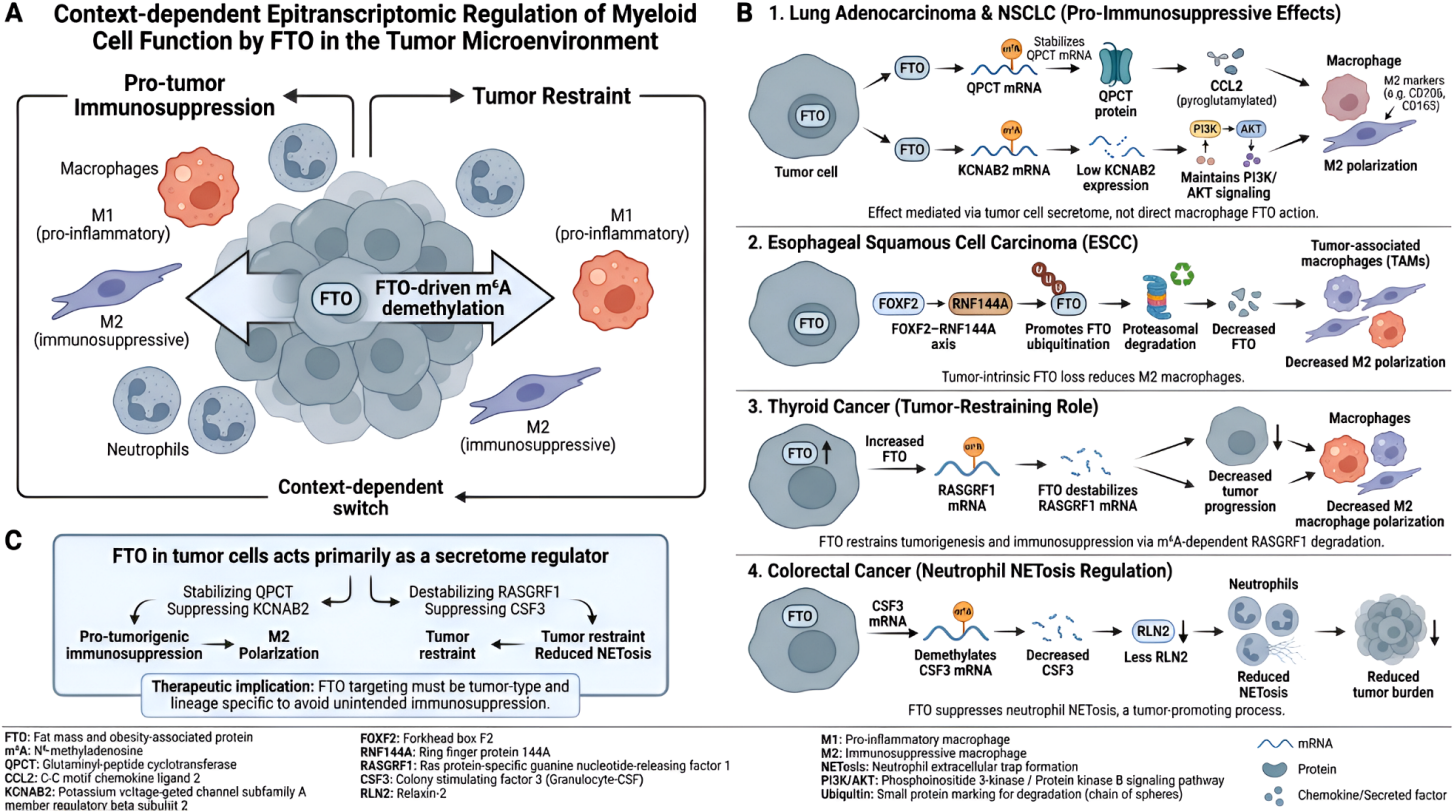
Tumor-cell-intrinsic FTO modulates the myeloid compartment through secretome-mediated effects on macrophages and neutrophils. Pathways shown: M2 polarization (LUAD/NSCLC, via QPCT or KCNAB2); reduced M2 polarization following FTO degradation (ESCC, FOXF2/RNF144A); RASGRF1-dependent restraint (thyroid); CSF3–RLN2-mediated NETosis suppression (CRC).

Collectively, these findings support a model in which FTO functions primarily as a tumor-cell-intrinsic regulator of the myeloid-modulating secretome, with its immunological consequences determined by the identity of stabilized or destabilized targets within each cancer type. This context-dependency presents both an opportunity and a hazard for therapeutic translation: lineage-specific and tumor-type-resolved FTO targeting strategies will be essential to avoid inadvertently promoting myeloid immunosuppression in settings where FTO inhibition paradoxically unleashes pro-tumorigenic myeloid programs (**Figure 3**).

### 3.4 Spatial architecture of immune exclusion

Immune exclusion, defined by the physical confinement of cytotoxic lymphocytes to stromal regions rather than tumor parenchyma, represents a clinically relevant barrier to immunotherapy efficacy. Emerging evidence implicates the epitranscriptome, and FTO specifically, as a potential mediator linking tumor-intrinsic m^6^;A programs to the structural and cellular determinants of this exclusion phenotype (**Table 2**).

At the physical level, cancer-associated fibroblasts (CAFs) function as the principal architects of immune exclusion in gastric cancer, deploying collagen-integrin interactions and MIF signaling to form stromal barriers that impede CD8^+^ T-cell entry into the tumor core (105). Critically, this biophysical barrier does not operate independently of epitranscriptomic regulation: spatially resolved analysis reveals that m^6^;A regulator scores are selectively elevated within tumor nests rather than surrounding stroma in immune-excluded samples, implicating epitranscriptomic activity as a spatially organized, tumor-compartment-specific program that reinforces stromal confinement (105). FTO is highly expressed within this immune-excluded phenotype and correlates with both stromal activation and T-cell exclusion, while the RNA-binding protein FMR1 sustains FTO protein abundance post-translationally through a proteasome-sensitive mechanism, forming an FMR1–FTO regulatory module that may stabilize the excluded state (105).

A critical interpretive limitation, however, must be acknowledged: the causal relationship between FTO enrichment and immune exclusion remains unestablished. FTO’s spatial enrichment in tumor nests may reflect the transcriptional state of an already-excluded niche rather than drive its formation, and the absence of functional FTO perturbation within spatially resolved systems precludes mechanistic conclusions. Whether the FMR1–FTO axis is upstream of CAF activation or secondary to it remains unresolved.

Nevertheless, these findings nominate the FMR1–FTO module as a candidate vulnerability at the intersection of epitranscriptomic and stromal programs. Spatially resolved functional perturbation combining FTO inhibition with CAF-targeting strategies represents a logical and urgently needed next step toward dismantling the structural and molecular foundations of immune exclusion.

## 4 FTO and immune checkpoint blockade

### 4.1 Direct regulation of checkpoint ligands by FTO

The regulation of PD-L1 by FTO operates through mechanistically distinct but functionally convergent layers, ranging from direct epitranscriptomic control of checkpoint mRNA to indirect amplification of oncogenic signaling cascades, a duality that renders FTO a context-sensitive modulator rather than a universal checkpoint driver (**Table 3**).

**Table 3:** FTO in tumor immune evasion and antitumor immunity: mechanistic, pharmacological, and clinical evidence across cancer types.

Cancer/model	FTO alteration/status	Key molecular axis	Immune effect	Cellular compartment	Evidence tier	Mechanistic category	Therapeutic/biological significance	Ref.
Breast cancer; hypoxic microenvironment; *in vitro* + mouse models	Upregulated (HIF-1α-transcriptionally induced); inhibited by FB23	HIF-1α → FTO↑ → PDK1 mRNA demethylation (↓YTHDF3 binding) → PDK1 stabilization → PDK1/AKT/STAT3 → PD-L1↑	PD-L1 upregulation; suppressed CTL activity	Tumor-intrinsic	ii	Indirect PD-L1 induction (hypoxia-dependent cascade)	FTO + PDK1 co-inhibition (FB23 + BX-912) enhances atezolizumab efficacy; sensitizes to PD-1/PD-L1 blockade	(107)
Colon cancer (HCT-116 cells); *in vitro*	Highly expressed; knocked down by siRNA	FTO directly demethylates PD-L1 mRNA; IFN-γ-independent mechanism confirmed by RIP	FTO knockdown reduces PD-L1 mRNA and protein; suppresses immune checkpoint expression	Tumor-intrinsic	iv	Direct PD-L1 mRNA regulation	Defines a candidate IFN-γ-independent m6A-based mechanism of PD-L1 regulation; single-cell-line evidence requiring orthogonal validation	(106)
Melanoma; *in vitro* + mouse models; human samples	Upregulated (induced by metabolic starvation via autophagy/NF-κB pathway); knocked down	FTO demethylates PDCD1 (PD-1), CXCR4, SOX10 → ↓YTHDF2-mediated RNA decay → protumorigenic gene stabilization	Reduced IFN-γ sensitivity; promotes anti-PD-1 blockade resistance; combination FTO-KD + anti-PD-1 increased CD4^+^ and CD8^+^ TIL densities and IFN-γ^+^ TIL frequencies (n = 9); sensitization abolished in NSG hosts, confirming adaptive immunity dependence	Tumor-intrinsic	ii	Multi-target checkpoint network destabilization	FTO knockdown sensitizes melanoma to anti-PD-1 blockade; FTO inhibition + anti-PD-1 as rational combination strategy	(23)
Hepatocellular carcinoma (HCC); *in vitro* + *in vivo*; patient samples	Upregulated in patient HCC tumors; knocked down; inhibited by CS2	FTO → m6A demethylation of GPNMB → GPNMB stabilization (↓YTHDF2-mediated degradation) → sEV-GPNMB → SDC4 on CD8+ T cells → T cell inhibition	CD8+ T cell activation and recruitment suppressed via sEV-GPNMB/SDC4 axis	Tumor-intrinsic (paracrine to immune cells)	ii	Exosomal immunosuppressive signaling	FTO inhibitor CS2 enhances anti-PD-1 and sorafenib efficacy; targeting FTO/m6A/GPNMB/sEV axis suppresses HCC growth and metastasis	(94)
Acute monocytic leukaemia (AML; THP-1 cells); *in vitro* + *in vivo* mouse xenograft; patient samples	Protein degraded by homoharringtonine (HHT; FDA-approved for CML)	HHT → FTO protein degradation → global m6A↑ → MLL1 and LILRB4 expression↓	LILRB4 downregulation enhances CD8+ T cell cytotoxicity; increased immune-activation markers	Tumor-intrinsic (paracrine to CD8^+^)	iii	Pharmacological FTO degradation (LILRB4 axis)	HHT repurposable as immunotherapy-sensitizing agent; LILRB4 downregulation overcomes immune evasion in monocytic AML	(96)
Melanoma (B16-F10; subcutaneous mouse model); nanodrug delivery	Silenced via FTO siRNA (tLyp-1-modified phase-transition nanoparticles co-loaded with Ce6)	FTO siRNA → glycolysis inhibition → lactate↓ → T cell activation barrier relieved; Ce6 + ultrasound → ROS → immunogenic cell death (ICD)	Enhanced DC and T lymphocyte infiltration; CD8+ T cell activation; ICD induction	Tumor-intrinsic (nanocarrier delivery)	ii	Nanotherapeutic delivery (FTO siRNA + sonodynamic)	FTO siRNA + sonodynamic therapy (SDT) reshapes immunosuppressive TME; synergistic antitumor efficacy in melanoma	(109)
Uveal melanoma (UM); orthotopic + metastatic *in vivo* models	Overexpressed (correlated with poor prognosis and aggressiveness); inhibited by meclofenamic acid (MA) loaded in SNAMA nanodrug + PD-L1 aptamer	MA/SNAMA → m6A↑ → SLC7A11 upregulation → GSH depletion + NADPH consumption → disulfidptosis	PD-L1 aptamer component enhances tumor targeting and immune modulation	Tumor-intrinsic (nanocarrier delivery)	ii	Nanotherapeutic delivery (disulfidptosis + checkpoint targeting)	FTO overexpression associated with UM aggressiveness and poor prognosis; SNAMA-apt induces disulfidptosis and enhances immune modulation; addresses bioavailability limitations of MA	(110)
Microsatellite-stable colorectal cancer (MSS-CRC); clinical samples + *in vitro*/*in vivo*	Elevated in CRC tissue; inhibited via cationic liposome co-delivering m6A modifier + CD47 siRNA	FTO inhibition → m6A↑ → lactate secretion↓ → M2-to-M1 TAM repolarization; CD47 siRNA → CD47↓ → enhanced M1-TAM phagocytosis	M1-like TAM polarization; enhanced macrophage phagocytosis; ‘cold’ TME converted towards immunogenic (‘hot’) phenotype	Tumor-intrinsic + Myeloid (TAM repolarization)	ii	Nanotherapeutic delivery (dual FTO + CD47)	Dual FTO + CD47 targeting overcomes immunotherapy resistance in MSS-CRC; strategy extendable to other immunologically cold tumors	(111)
Esophageal squamous cell carcinoma (ESCC); TCGA + GEO cohorts (n = 453); bioinformatic	NR (FTO-specific expression or functional data not reported among key results)	PD-L1 negatively correlated with YTHDF2, METTL14, and KIAA1429; m6A regulators linked to immune cell infiltration and TIME clusters	Cluster 2 associated with higher CD8+ T cells, Tregs, and immune score; m6A regulators influence TIME composition	Bulk tissue (cellular source not resolvable)	v	Multi-gene m6A signature (correlative)	Five-gene m6A prognostic signature independently predicts ESCC outcome; m6A regulators modulate PD-L1 and TIME; FTO-specific immune role NR	(108)
Multiple solid cancers (retrospective; n = 371; ICI-treated patients)	Germline polymorphisms genotyped: rs11075995, rs1125338, rs12596638, rs12600192	FTO germline variants associated with PFS, OS, and irAE risk under ICI treatment; mechanistic basis NR	NR (germline pharmacogenomic study; no cellular immune mechanism reported)	Germline (host genetic; not cell-intrinsic)	v	Germline pharmacogenomic biomarker	rs12596638GG associated with extended PFS; rs12600192CC associated with improved OS and lower severe irAE risk; FTO SNPs as candidate pharmacogenomic ICI biomarkers	(112)
Colorectal cancer (CRC); TCGA + GEO (n = 1,047); machine learning	Included among 8 m6A regulators selected by LASSO regression; individual FTO effect not distinguished	Eight-gene m6A signature (incl. FTO) for risk stratification; Random Forest model (training AUC = 0.895; validation AUC = 0.847); primary SHAP contributors: IGF2BP2, METTL3	Low-risk patients: higher CD8+ T cell infiltration (17.8% vs. 10.2%) and better predicted ICI response (36.5% vs. 20.3%)	Bulk tissue (cellular source not resolvable)	v	Multi-gene m6A signature (FTO not isolated)	Eight-gene m6A signature predicts CRC prognosis and ICI response; FTO individual prognostic contribution not isolated by SHAP analysis	(114)
Acute myeloid leukaemia (AML); TCGA + GEO; bioinformatic (WGCNA + LASSO)	NR (FTO not identified among the defining hub genes EHBP1L1 or ZNF385A of the risk model)	m6A-immune risk model (EHBP1L1, ZNF385A); TME correlations with monocytes and Treg cells; immune checkpoint and HLA gene associations with risk score	High-risk patients: Treg and monocyte enrichment; reduced predicted ICI benefit (TIDE analysis)	Bulk tissue (cellular source not resolvable)	v	Multi-gene m6A-immune risk model (FTO not in hub)	m6A-immune risk model predicts AML prognosis and guides chemotherapy selection; FTO-specific role in AML immune landscape NR	(115)

Cellular compartment indicates the cell type in which FTO is acting: *Tumor-intrinsic*, FTO functions within tumor cells; *Germline*, host-genotype-level effect (not cell-intrinsic); *Bulk tissue*, correlative analyses where the cellular source of FTO signal is not resolvable.

Evidence tier (per the Evidence Calibration framework in Section 2.1): (i) mechanisms validated by genetic perturbation in multiple *in vivo* models with patient-sample correlation; (ii) single *in vivo* perturbation study with mechanistic support; (iii) multi-cell-line *in vitro* evidence; (iv) single-cell-line or single-model evidence; (v) correlative bioinformatic or transcriptomic evidence only.

Mechanistic category groups studies by primary mechanism (direct vs. indirect PD-L1 regulation, multi-target network, exosomal signaling, pharmacological degradation, nanotherapeutic delivery, transcriptomic biomarker, germline pharmacogenomic) to facilitate cross-study pattern recognition.

Pattern note. Across **Table 3**, the evidence base for FTO–checkpoint regulation falls into three distinct strata. (i) Mechanistic studies with *in vivo* validation define multiple non-equivalent routes by which tumor-intrinsic FTO can influence checkpoint biology, direct PD-L1 mRNA regulation, hypoxia-gated PDK1/AKT/STAT3 induction, multi-target network destabilization (PDCD1/CXCR4/SOX10), and exosomal GPNMB signaling, without converging on a single dominant mechanism. (ii) The widely cited “direct PD-L1 demethylation” finding in colon cancer rests on Tier iv evidence (single cell line, HCT-116, *in vitro* only) and should be interpreted as a candidate mechanism pending orthogonal genetic and *in vivo* validation, particularly given that the breast cancer study, and HCC studies demonstrate substantively different routes to PD-L1 modulation. (iii) Five of thirteen rows are Tier v bioinformatic analyses in which FTO is either correlated with checkpoint expression in bulk transcriptomes, embedded in multi-gene m6A signatures where its individual contribution cannot be isolated, or, in two cases, not identified as a hub gene at all. This distribution underscores that the conceptual case for FTO–ICI combination therapy rests predominantly on Tier ii–iii preclinical evidence, with Tier v correlative work providing supporting but not independent rationale.↑, increased expression, activation, or upregulation; ↓, decreased expression, inhibition, downregulation, or loss; →, mechanistic or regulatory relationship between molecular events.

The most parsimonious regulatory mechanism is direct: FTO physically binds PD-L1 mRNA in colon cancer cells, and its demethylation activity stabilizes the transcript, sustaining PD-L1 protein expression independently of canonical IFN-γ signaling (106). These findings suggest that FTO may contribute to immune evasion independently of inflammatory stimulation, and that epitranscriptomic stabilization of PD-L1 could represent a baseline immune-escape mechanism in FTO-high tumors. This conclusion, however, rests almost entirely on siRNA-mediated knockdown in a single cell line (HCT-116), without orthogonal validation in primary human tumors or *in vivo* models, a limitation that materially constrains causal inference.

A mechanistically distinct but functionally convergent route operates in breast cancer under hypoxia, where HIF-1α-induced FTO stabilizes PDK1 mRNA via demethylation-dependent escape from YTHDF3-mediated decay, ultimately driving AKT/STAT3-dependent PD-L1 transcription (107). The hypoxia dependency of this axis introduces contextual specificity, suggesting it may operate predominantly within hypoxic tumor niches rather than across the entire tumor, and is discussed in further integrative detail in the “Metabolic-Immune Coupling” section.

At the systems level, comprehensive m^6^;A regulator profiling in esophageal squamous cell carcinoma identifies PD-L1 as negatively correlated with specific m^6^;A writers and readers across molecular subtypes, and associates m^6^;A regulator clusters with distinct immune infiltration patterns (108). While these correlative findings implicate m^6^;A programs broadly in checkpoint regulation and TIME composition, they cannot resolve whether FTO specifically drives these associations or whether they reflect the combined activity of the entire m^6^;A machinery.

**Figure 4:**
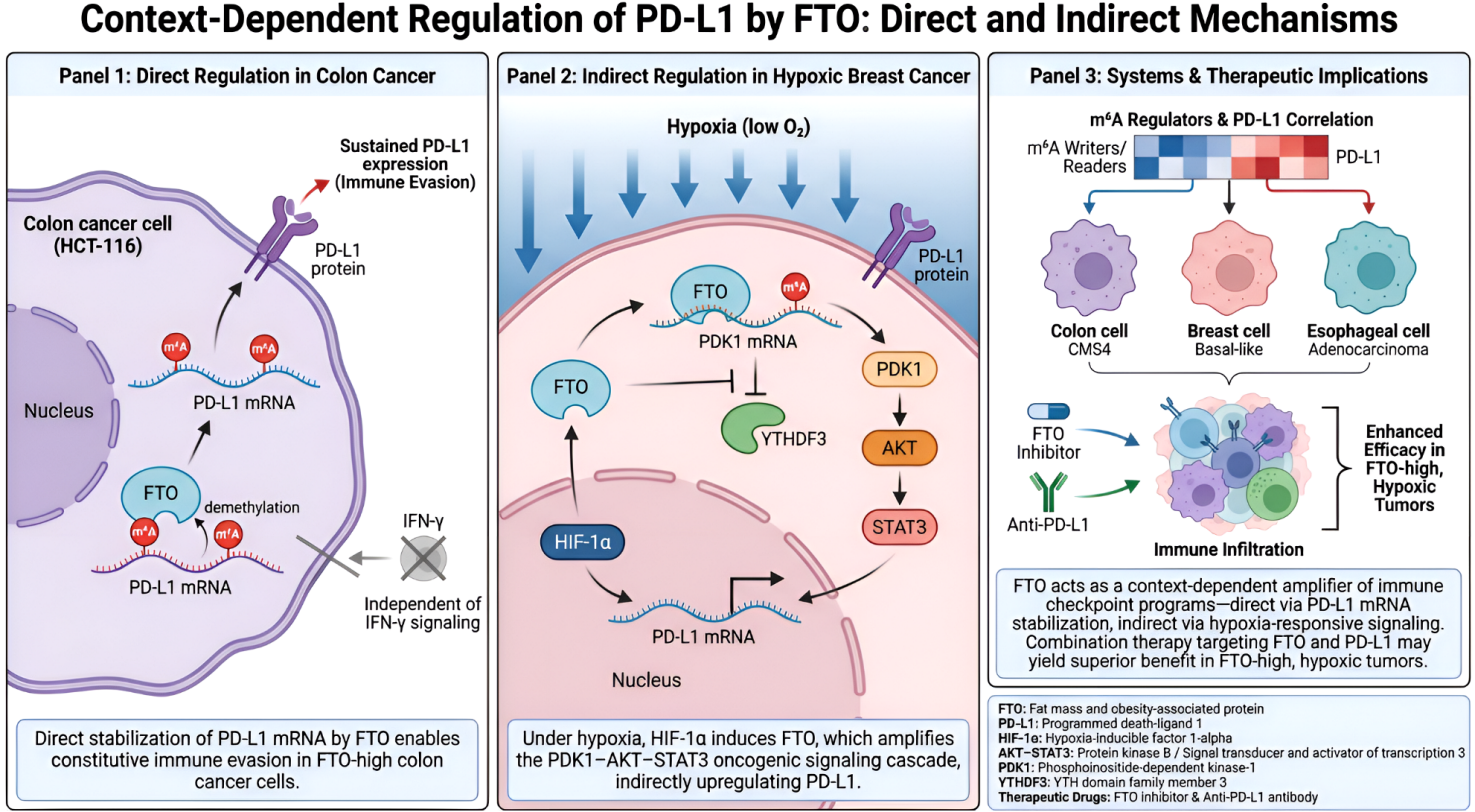
Two distinct mechanisms by which tumor-cell-intrinsic FTO has been linked to PD-L1 expression: direct m^6^;A-dependent stabilization of PD-L1 mRNA in colon cancer cells (left) and indirect activation via the HIF-1α–FTO–PDK1–AKT–STAT3 axis under hypoxia in breast cancer (right). the colon cancer evidence derives from a single cell-line model (HCT-116).

Collectively, the available evidence supports a model in which FTO functions as a context-dependent amplifier of checkpoint programs direct in its post-transcriptional stabilization of PD-L1 mRNA, and indirect as a hypoxia-responsive transducer of oncogenic signaling. This context-sensitivity has immediate therapeutic implications: combining FTO inhibition with checkpoint blockade may be most efficacious in hypoxic, FTO-high tumors, while pan-tumor application risks incomplete checkpoint suppression in contexts where PD-L1 regulation is signaling-dominated rather than epitranscriptomically driven (**Figure 4**).

### 4.2 *In Vivo* evidence and combination nanotherapeutics

The Yang et al. melanoma study (23) is a useful exemplar of how tumor-cell-intrinsic FTO perturbation can produce immune-compartment consequences, and we discuss it accordingly: the FTO knockdown described is restricted to tumor cells, and the observed CD4^+^ and CD8^+^ TIL changes are downstream readouts of that tumor-intrinsic perturbation rather than measurements of FTO activity within the lymphocytes themselves. Preclinical investigation of FTO’s mechanistic roles in immune evasion has advanced, with converging *in vivo* evidence and nanotherapeutic platforms of increasing complexity, supporting FTO as a candidate regulatory node linking tumor metabolic reprogramming, immune exclusion, and resistance to immunotherapy. Yet a critical appraisal of this body of work reveals both the promise and the considerable complexity of targeting FTO in the clinical setting (**Table 3**).

Beyond bioinformatic correlations, a single direct tumor-context observation has been reported: in the B16F10 melanoma model, tumor-intrinsic FTO knockdown combined with anti-PD-1 blockade increased CD4^+^ tumor-infiltrating lymphocyte densities in parallel with CD8^+^ TIL increases (23), indicating that FTO-mediated immune evasion in melanoma constrains both lymphoid effector arms. However, this finding reflects FTO perturbation in the tumor compartment rather than in CD4^+^ T cells themselves, and therefore does not establish CD4^+^-intrinsic FTO function. This finding provides mechanistic evidence that FTO inhibition may reduce checkpoint ligand expression while also affecting a broader network of m^6^;A-sensitive pro-evasion transcripts, potentially altering the tumor’s immune contexture.

In AML, the pharmacological dimension is reinforced by HHT-induced FTO proteasomal degradation, which suppresses LILRB4 and MLL1, enhancing CD8^+^ T-cell cytotoxicity in xenograft models and patient-derived samples (96). In the hydrodynamic tail-vein Trp53KO/C-MycOE HCC model, pharmacological inhibition of FTO with CS2 reduced liver weight, used as a surrogate of tumor burden, by approximately 50–55% relative to control. Combination treatment with CS2 plus anti-PD-1 produced a further reduction of approximately 20–25% relative to CS2 alone and approximately 55–60% relative to anti-PD-1 alone. Consistent with this enhanced tumor suppression, tumor-infiltrating CD8a^+^ T cells increased by approximately fourfold relative to control and by approximately 1.8-fold relative to CS2 monotherapy under the combination regimen. Mechanistically, the study linked this immunologic effect to the FTO–m^6^;A–GPNMB–sEV axis: FTO stabilized GPNMB mRNA by reducing m^6^;A-dependent YTHDF2-mediated degradation, while HCC-derived sEV-associated GPNMB bound SDC4 on CD8^+^ T cells and suppressed their activation. GPNMB knockdown, or blockade of SDC4, relieved this suppression of CD8^+^ T-cell activation, supporting, but not by itself fully proving, the sEV-GPNMB pathway as a functional mediator of FTO-associated immune evasion (94), indicating that a single epitranscriptomic intervention may disrupt multiple, mechanistically distinct immune evasion pathways.

The metabolic dimension of FTO-targeted therapy is most explicitly addressed in nanotherapeutic platforms. In melanoma, peptide-modified phase-transition nanoparticles co-delivering FTO siRNA and the sonosensitizer Ce6 leverage FTO inhibition to suppress glycolysis and reduce lactate accumulation, thereby removing a key metabolic barrier to T-cell and dendritic cell activation, while SDT-induced immunogenic cell death provides a complementary immunostimulatory signal (109). This dual-modality design is predicated on metabolic and immunogenic reprogramming acting complementarily rather than redundantly. A parallel logic governs the uveal melanoma SNAMA platform, in which meclofenamic acid-loaded nucleic acid nanodrugs restore m^6^;A levels to induce disulfidptosis via SLC7A11 upregulation, while PD-L1 aptamer incorporation provides tumor-selective delivery and direct checkpoint modulation (110). In MSS colorectal cancer a classically immunologically cold tumor cationic liposomes co-delivering FTO-targeting cargo and CD47 siRNA reduce lactate secretion to repolarize M2 TAMs toward an M1 phenotype while simultaneously disabling the “don’t-eat-me” signal, converting a suppressive myeloid niche into an active phagocytic environment (111).

Accordingly, these nanotherapeutic platforms should be understood as preclinical proof-of-concept demonstrations of mechanistic rationale, not as near-term clinical interventions. None has yet entered formal IND-enabling development, and pharmacokinetic, biodistribution, and safety datasets in non-rodent species, prerequisites for clinical translation, have not been reported. Nanotherapeutic platforms differ substantially in their delivery chemistry, targeting moieties, and release kinetics, making mechanistic comparison across systems difficult and raising unresolved questions about off-target FTO inhibition in normal tissues particularly lymphoid compartments where FTO supports CD8^+^ T-cell survival, as established in earlier work. Whether nanodrug platforms fundamentally alter biological response or primarily optimize pharmacokinetic access to known mechanisms remains an open and underappreciated question.

Collectively, these preclinical findings indicate that FTO inhibition can disrupt metabolic, exosomal, and checkpoint axes of immune evasion in parallel. Tumor-selective, lineage-aware FTO-targeting strategies, guided by spatial and single-cell mapping of FTO activity in human tumors, will need to be developed and clinically validated before therapeutic potential can be assessed.

### 4.3 Predictive biomarkers: from germline to tumor transcriptome

Predictive biomarkers for immunotherapy response remain a major unmet clinical need. The m^6^;A regulatory axis, including FTO, has been proposed as a candidate biomarker framework spanning germline variation, tumor transcriptomics, and immune landscape composition (**Table 3**). At the germline level, pharmacogenomic interrogation of FTO single-nucleotide polymorphisms in a pan-cancer cohort of ICI-treated patients reveals that specific FTO variants independently associate with progression-free survival, overall survival, and severe immune-related adverse events (112). The rs12596638GG genotype confers extended survival while rs1125338TT predicts shorter PFS, and rs12600192CC correlates with reduced toxicity risk findings that collectively nominate germline FTO variation as a pre-treatment predictive signal (112). Critically, however, this study is retrospective (112), encompasses heterogeneous cancer types without stratification, and provides no mechanistic linkage between these polymorphisms and FTO protein function, m^6^;A levels, or immune phenotype. The associations therefore remain correlative surrogates rather than mechanistically anchored predictors.

At the tumor-transcriptomic level, elevated FTO expression in gastric cancer has been associated with an immunologically cold subgroup characterized by depleted CD8^+^ T-cell infiltration, enhanced stemness, high tumor immune dysfunction and exclusion (TIDE) scores, and negative correlation with both tumor mutational burden and microsatellite instability, alongside positive correlation with PD-L1 (113). The negative correlation with microsatellite instability is of particular interest, since microsatellite-unstable tumors are the population most likely to benefit from current immune checkpoint inhibitor regimens; FTO-high gastric tumors may therefore represent a candidate biomarker for predicted checkpoint-inhibitor resistance. In colorectal cancer, a machine-learning model integrating eight m^6^;A regulators (including FTO) achieves prognostic discrimination across independent cohorts and stratifies patients by predicted immunotherapy response, with low-risk patients showing higher CD8^+^ T-cell infiltration and response rates (114). A parallel m^6^;A-immune risk model in acute myeloid leukemia correlates with monocyte and regulatory T-cell infiltration, checkpoint-gene expression, and TIDE-predicted resistance to checkpoint blockade (115).

Collectively, these findings outline a candidate multi-layer biomarker model: germline FTO variants, tumor-level FTO overexpression, and composite m^6^;A signatures together correlate with immune infiltration patterns and predicted ICI response. Yet critical limitations pervade all layers. All transcriptomic analyses rely on bulk TCGA/GEO data, which conflate tumor cell, stromal, and immune contributions without spatial or cellular resolution. FTO’s biomarker value may therefore reflect composite TME states rather than epitranscriptomic biology per se a distinction with profound implications for mechanistic interpretation. Prospective validation across standardized cohorts receiving defined ICI regimens is entirely absent, and cancer-type heterogeneity limits generalizability.

Advancing FTO-centered biomarker models toward clinical utility will require multi-omics integration incorporating germline, epigenomic, single-cell transcriptomic, and spatially resolved immune data to distinguish true drivers of immune responsiveness from surrogate signals of broader tumor biology.

## 5 FTO and therapeutic resistance

### 5.1 Cytotoxic chemotherapy resistance: convergent death-pathway evasion

A clinically important function of FTO-mediated m^6^;A demethylation is its capacity to reshape the tumor cell’s regulated-cell-death network, the balance of apoptotic and regulated-death pathways that determines survival under chemotherapeutic insult. Critically, FTO does not operate as a unidirectional pro-survival enzyme but as a context-sensitive epitranscriptomic switchboard whose polarity is determined by target transcript identity, cancer type, and the specific drug employed (**Table 4**).

**Table 4:** Role of FTO in Therapy Response and Resistance Across Cancer Types: Chemotherapy, Targeted Therapy, Radiotherapy, and Intercellular Transmission.

Cancer/biological context	Therapy/resistance context	FTO status/perturbation	Main mechanistic axis	Cellular compartment	Evidence tier	Direction & mechanistic category	Key conclusion	Ref.
Non-malignant, toxicity-protective contexts (FTO as cytoprotective agent — not tumor chemoresistance)								
Ovarian granulosa cells (KGN); cisplatin-induced POF model	Cisplatin gonadotoxicity protection (non-malignant)	Downregulated by cisplatin; overexpressed/silenced experimentally	FTO → Hippo/YAP1 inhibition → YAP1 nuclear entry → CTGF/CYR61/ANKRD1↑	Non-malignant (granulosa)	iii	Cytoprotective (apoptosis suppression)	FTO overexpression protects granulosa cells from cisplatin-induced apoptosis via Hippo/YAP1; FTO silencing exacerbates apoptosis; protective role in non-malignant POF model; *in vitro*	(129)
Ovarian granulosa cells (KGN); cisplatin-induced POF model	Cisplatin toxicity; FGF2-mediated cytoprotection	Downregulated in cisplatin-injured cells; rescued by exogenous FGF2	FGF2 → FTO↑ → autophagy activation → apoptosis suppression	Non-malignant (granulosa)	iii	Cytoprotective (autophagy)	FGF2 attenuates cisplatin-induced granulosa cell apoptosis by upregulating FTO and activating autophagy; non-malignant context only; *in vitro*	(130)
Cardiomyocytes (H9C2 + mouse hearts); doxorubicin cardiotoxicity	Doxorubicin cardiotoxicity; cardioprotection (non-malignant)	Downregulated by doxorubicin; overexpressed experimentally	FTO → P53 m6A demethylation → P53/P21/Nrf2 axis → ferroptosis inhibition; HuR-FTO-P53-P21 feedback loop	Non-malignant (cardiomyocyte)	ii	Cytoprotective (ferroptosis suppression)	FTO overexpression protects cardiomyocytes from doxorubicin ferroptosis via P53-P21/Nrf2/HuR axis; non-malignant cardiotoxicity model; does not represent tumor chemoresistance; *in vitro* + *in vivo*	(128)
Solid tumor chemoresistance — FTO upregulated/oncogenic (FTO promotes resistance)								
Pancreatic cancer; gemcitabine-resistant lines + patient tissues	Gemcitabine resistance	Upregulated in GR cells/tissues; USP7-stabilized; knocked down	USP7 → FTO stabilization → NEDD4 m6A demethylation (YTHDF2) → NEDD4↑ → PTEN↓ → PI3K/AKT activation	Tumor-intrinsic	ii	FTO promotes resistance (PI3K/AKT axis)	FTO promotes gemcitabine resistance via NEDD4/PTEN/PI3K/AKT axis; FTO depletion inhibits GR tumor growth *in vivo*; *in vitro* + *in vivo* + clinical tissues	(120)
PDAC; cell lines + primary patient tumors	Gemcitabine (GEM) resistance	Functionally stabilizes LINC01134 via m6A demethylation	FTO → LINC01134 stabilization (YTHDF2-dependent) → miR-140-3p↓ → WNT5A↑ → WNT activation → stemness	Tumor-intrinsic	ii	FTO promotes resistance (lncRNA/Wnt)	FTO-mediated LINC01134 stabilization promotes GEM resistance via miR-140-3p/WNT5A/WNT pathway; LINC01134 identified as prognostic marker; *in vitro* + *in vivo* + patient tumors	(121)
CRC; CRISPR/Cas9 cellular and mouse models	Chemoresistance (agent not reported)	Upregulated; CRISPR/Cas9 knockout	FTO → NUPR1 m6A demethylation (+451 site; ↓YTHDF2 decay) → NUPR1↑ → LCN2/FTH1↑ → iron homeostasis dysregulation	Tumor-intrinsic	ii	FTO promotes resistance (iron homeostasis)	FTO stabilizes NUPR1 to drive CRC chemoresistance through iron homeostasis regulation; simultaneous FTO+NUPR1 targeting enhances chemotherapy efficacy; *in vitro* + *in vivo* (CRISPR mouse model)	(20)
CRC; 5-FU-resistant lines + patient tissues	5-Fluorouracil (5-FU) resistance	Upregulated in 5-FU-resistant CRC vs. naive primary CRC	FTO → SIVA1 CDS m6A demethylation → SIVA1 mRNA decay (YTHDF2) → pro-apoptotic SIVA1 suppression	Tumor-intrinsic	ii	FTO promotes resistance (apoptosis suppression)	FTO promotes 5-FU resistance by silencing SIVA1-mediated apoptosis; FTO inhibition restores 5-FU sensitivity; SIVA1 inversely correlated with FTO in clinical tissues; *in vitro* + *in vivo* + clinical	(116)
CRC; 5-FU-resistant cells + PDOs + xenograft + clinical cohort	5-Fluorouracil (5-FU) resistance; CS1 inhibitor	Upregulated in CRC vs. NCM460; inhibited by CS1 (FTO inhibitor)	FTO↑ → EMT activation, stemness↑, p-AKT/PCNA↑, Caspase-9↓; CS1 → FTO↓ → 5-FU sensitization	Tumor-intrinsic	ii	FTO promotes resistance (EMT/stemness)	FTO overexpression promotes 5-FU resistance and aggressiveness in CRC; CS1 sensitizes CRC cells and PDOs to 5-FU; FTO upregulation correlates with poor OS and RFS; *in vitro* + xenograft + PDOs + clinical cohort	(117)
Gastric cancer; cell lines + patient tissues	5-Fluorouracil (5-FU) resistance	Upregulated in GC cells/tissues	FTO → CDKAL1 m6A demethylation → CDKAL1↑ → mitochondrial fusion → proliferation + 5-FU resistance	Tumor-intrinsic	iii	FTO promotes resistance (mitochondrial dynamics)	FTO promotes 5-FU resistance in GC by upregulating CDKAL1 and inducing mitochondrial fusion; *in vitro* + patient tissue	(122)
Gastric cancer; cell lines + TCGA	5-FU, cisplatin, paclitaxel; omeprazole as chemosensitizer	Upregulated in GC (TCGA); suppressed by omeprazole	Omeprazole → FTO↓ → global m6A↑ → mTORC1 activation → autophagy suppression + DDIT3↑ → drug cytotoxicity↑	Tumor-intrinsic	iii	FTO promotes resistance (mTORC1/autophagy)	Omeprazole-induced FTO suppression enhances GC sensitivity to multiple chemotherapeutics via mTORC1/DDIT3 axis; FTO upregulation negatively correlates with DFS in TCGA; *in vitro* + TCGA	(123)
Breast cancer (BC-DoxR cell lines)	Doxorubicin resistance	Upregulated in BC-DoxR; STAT3-transactivated (ChIP-validated); KD/OE	STAT3 → FTO promoter binding → FTO transcription↑ → m6A regulation + STAT3 pathway activation (positive feedback)	Tumor-intrinsic	iii	FTO promotes resistance (STAT3 feedback)	STAT3-driven FTO upregulation promotes doxorubicin resistance via STAT3-FTO positive feedback loop; FTO knockdown reduces resistance; *in vitro* only	(118)
Breast carcinoma; docetaxel-resistant lines	Docetaxel resistance	Functionally promotes LINC01559 via m6A demethylation	FTO → LINC01559 m6A demethylation (YTHDF2) → LINC01559 stability↑ → miR-1343-3p suppression	Tumor-intrinsic	iii	FTO promotes resistance (lncRNA stabilization)	FTO-mediated epigenetic stabilization of LINC01559 confers docetaxel resistance in BCa via miR-1343-3p suppression; *in vitro* only	(119)
NSCLC; gefitinib-resistant lines	Gefitinib resistance	Upregulated in gefitinib-resistant NSCLC	FTO↑ → PELI3↑ + autophagy activation → gefitinib resistance	Tumor-intrinsic	ii	FTO promotes resistance (autophagy)	FTO promotes gefitinib resistance via PELI3 upregulation and autophagy in NSCLC; PELI3/autophagy manipulation reverses resistance; *in vitro* + *in vivo*	(132)
DLBCL; xenograft + patient samples	Ibrutinib resistance	Upregulated in DLBCL patient samples and lines; KO/KD/OE	FTO → MYC m6A demethylation → MYC mRNA stability↑ → MYC protein↑ → growth + ibrutinib resistance	Tumor-intrinsic	ii	FTO promotes resistance (MYC stabilization)	FTO promotes ibrutinib resistance and DLBCL progression by stabilizing MYC; FTO-m6A-MYC axis as therapeutic target; *in vitro* + xenograft + patient samples	(143)
AML; CR vs relapse samples + cell lines	Cytosine arabinoside (Ara-C); relapse-associated chemoresistance	Overexpressed in AML relapse vs CR (MeRIP-seq)	FTO at relapse → FOXO3 m6A demethylation → FOXO3 mRNA destabilization → impaired myeloid differentiation	Tumor-intrinsic	ii	FTO promotes resistance (differentiation block)	FTO overexpression at relapse promotes Ara-C resistance by destabilizing FOXO3 and impairing differentiation; FTO as therapeutic target for AML relapse; *in vitro* + *in vivo* + sequential clinical samples	(145)
ESCC; cell lines	Chemoresistance (agent not reported)	Functionally stabilizes LINK-A via m6A demethylation	FTO → LINK-A stabilization → MCM3-CDK1 complex → MCM3 phosphorylation → cell cycle + HIF-1α de-repression → glycolysis↑	Tumor-intrinsic	iii	FTO promotes resistance (lncRNA/HIF-1α)	FTO stabilizes LINK-A to drive cell cycle progression and chemoresistance via MCM3/HIF-1α axis; chemotherapy agent not specified; *in vitro* only	(146)
DLBCL; multiple cell lines	Cancer aggressiveness (no specific drug resistance phenotype)	Upregulated in DLBCL cell lines vs WIL2S; OE/silencing	FTO → FLOT2 m6A demethylation → FLOT2 stability↑ → p-PI3K/p-Akt/p-mTOR activation	Tumor-intrinsic	iii	FTO promotes aggressiveness (PI3K/Akt/mTOR)	FTO promotes DLBCL aggressiveness via FLOT2/PI3K/Akt/mTOR axis; therapy-resistance context not explicitly studied; *in vitro* only	(144)
FTO loss or inhibition sensitizes tumor cells/FTO downregulation associated with resistance								
Ovarian cancer; cisplatin-resistant vs sensitive lines + tissues	Cisplatin resistance; FTO restores sensitivity	Downregulated in OC tissues and DDP-resistant cells; OE	FTO → NLRP3 m6A demethylation → NLRP3/Caspase-1/GSDMD pyroptosis → DDP IC50↓	Tumor-intrinsic	ii	FTO opposes resistance (pyroptosis activation)	FTO overexpression restores cisplatin sensitivity in resistant OC by activating NLRP3/Caspase-1/GSDMD pyroptosis; *in vitro* + xenograft + clinical tissues	(127)
Bladder urothelial carcinoma; TCGA	Cisplatin cytotoxicity; MA2 inhibitor (paradoxical)	Downregulated in BUC vs normal (PCR+TCGA); MA2 used pharmacologically	FTO loss → oncogenic phenotype; MA2 inhibits FTO → reduces cisplatin cytotoxicity (paradoxical)	Tumor-intrinsic	iii	FTO opposes resistance (drug efflux; paradoxical)	FTO downregulation oncogenic in BUC; MA2-mediated FTO inhibition reduces cisplatin cytotoxicity, suggesting FTO activity required for cisplatin sensitivity; *in vitro* + TCGA	(125)
NSCLC; cisplatin-resistant (CR) tissues and cells	Cisplatin resistance	Downregulated in CR NSCLC vs sensitive	FTO loss → 5’-UTR m6A↑ on resistance mRNAs (ATP7A, ERCC1, CD99, CDKN3, XRCC5, NOL3) → eIF3A recruitment impaired → translation silencing; RBM5-FTO interaction	Tumor-intrinsic	iii	FTO opposes resistance (translation regulation)	FTO loss in CR NSCLC impairs translation of resistance-modulating mRNAs via 5’-UTR m6A/RBM5/eIF3A axis; FTO functions as translational regulator in this context; *in vitro* + clinical tissues	(124)
NSCLC (A549); paclitaxel-resistant lines	Paclitaxel resistance; entacapone (FTO inhibitor)	FTO knockdown or entacapone sensitizes to PTX	FTO inhibition → MRP7 mRNA m6A↑ → YTHDC2/YTHDF2 decay → MRP7↓ → intracellular PTX↑	Tumor-intrinsic	iii	FTO promotes resistance (drug efflux)	FTO knockdown or entacapone enhances PTX sensitivity in NSCLC by reducing MRP7-dependent drug efflux via m6A-dependent MRP7 mRNA regulation; *in vitro* only	(126)
NSCLC; gefitinib-resistant lines	Gefitinib resistance; meclofenamic acid (MA)	Inhibited by MA (NSAID used as FTO inhibitor)	MA → FTO inhibition → global m6A↑ → c-Myc↓ → BCRP/MRP7↓ → intracellular GE↑ → apoptosis	Tumor-intrinsic	iii	FTO promotes resistance (drug efflux via c-Myc)	MA restores gefitinib sensitivity in resistant NSCLC by suppressing BCRP/MRP7 drug efflux via FTO/m6A/c-Myc axis; synergistic effect confirmed by Chou-Talalay method; *in vitro* only	(133)
Pan-cancer epithelial; PDX + clinical datasets	Wnt inhibitor sensitivity; EMT-mediated progression	Downregulated across multiple epithelial cancers (widespread)	FTO loss → m6A↑ → altered 3’-end processing of Wnt mRNAs → EMT activation → invasion/metastasis↑	Tumor-intrinsic	ii	FTO opposes resistance (EMT regulation)	Widespread FTO downregulation promotes EMT and worse outcome across epithelial cancers; FTO-low tumors sensitized to Wnt inhibitors; *in vitro* + PDX + clinical datasets	(134)
ccRCC; HIF2α-low subtype; CRISPR screen + PDX	HIF2α antagonist resistance; sensitivity to BRD9 inhibitor I-BRD9	Aberrantly activated in HIF2α-low ccRCC (↑α-KG/succinate ratio)	α-KG/succinate↑ → FTO activation → BRD9 m6A demethylation → BRD9 stabilization → SOX17-BRD9 super-enhancers → CCND1/VEGFR2/CDC20/SRC/MAPK6↑	Tumor-intrinsic	ii	FTO promotes resistance (super-enhancer)	Aberrant FTO activation drives BRD9-dependent oncogenesis in HIF2α-low ccRCC; I-BRD9 suppresses tumor growth (greater efficacy than sunitinib); *in vitro* + xenograft + PDX	(135)
Malignant glioma (U87)	Temozolomide (TMZ) sensitivity; MA2 (ethyl ester of MA)	Promotes glioma growth; MA2 inhibits	FTO → MYC m6A demethylation → MYC↑ → miR-155/miR-23a cluster↑ → MXI1↓; MXI1 suppresses MYC (negative feedback); MA2 disrupts loop	Tumor-intrinsic	iii	FTO promotes resistance (MYC feedback loop)	FTO sustains oncogenic MYC-miR-155/23a-MXI1 feedback loop in glioma; MA2 disrupts loop and synergistically enhances TMZ efficacy; *in vitro* only	(139)
GBM; TMZ-resistant lines	TMZ resistance; SYSUP007 (novel rhein derivative)	Promotes TMZ resistance; inhibited by SYSUP007 dose-dependently	FTO → SOX9 m6A demethylation → SOX9↑ → TMZ resistance; SYSUP007 → FTO↓ → m6A↑ → SOX9↓ → TMZ sensitization	Tumor-intrinsic	iii	FTO promotes resistance (SOX9 stabilization)	FTO stabilizes SOX9 to drive TMZ resistance in GBM; SYSUP007 synergistically enhances TMZ efficacy via FTO/m6A/SOX9 axis; *in vitro* only	(138)
Malignant glioma (U251, U87, A172)	TMZ sensitivity; PDC (Phosducin) downstream	FTO negatively regulates PDC; OE/KD	FTO↑ → m6A-dependent PDC suppression; PDC OE → proliferation↓ + TMZ efficacy↑	Tumor-intrinsic	iii	FTO promotes resistance (via PDC)	FTO suppresses PDC to impair TMZ sensitivity in glioma; PDC overexpression enhances TMZ efficacy; *in vitro* only	(140)
Neuroblastoma; cell lines + patient cohort	Drug-dependent (etoposide, paclitaxel, cisplatin)	High FTO correlates with favorable prognosis; OE/KD	FTO effects drug-specific: etoposide sensitivity↑; cisplatin no effect; paclitaxel mixed; mechanism not detailed	Tumor-intrinsic	iv	FTO direction is drug-specific	FTO diversely modulates NB chemosensitivity in drug-dependent manner; high FTO correlates with improved prognosis; mechanistic detail not reported; correlative + *in vitro*	(131)
Radiotherapy and combined chemo-radiotherapy response								
HPV-negative HNSCC; human + mouse xenograft	Radiotherapy resistance; FTO inhibition enhances therapeutic index	Inhibited genetically and pharmacologically	FTO inhibition → DNA damage↑ → homologous recombination↓ (RAD51 foci↓) → radiation-induced cell kill↑	Tumor-intrinsic	ii	FTO promotes resistance (homologous recombination)	FTO inhibition enhances RT efficacy in HPV-negative HNSCC by impairing HR DNA repair; does not worsen oral mucositis; *in vitro* + xenograft	(136)
Glioblastoma stem cells; *in situ* intracranial mouse model	Radiotherapy resistance; FB23–2 inhibitor	Promotes radioresistance and stemness; inhibited by FB23-2	FTO↑ → VEGFA m6A demethylation → VEGFA↑ + GSC stemness; FB23-2 + RT → γ-H2AX↑, Rad51↓	Tumor-intrinsic	ii	FTO promotes resistance (stemness + DNA damage)	FTO promotes GSC radioresistance and stemness via VEGFA stabilization; FB23-2 + RT inhibits intracranial tumour growth and prolongs survival; *in vitro* + *in situ* mouse model	(137)
CSCC; cell lines + patient tissues	Combined chemo-radiotherapy resistance	Upregulated in CSCC vs adjacent normal	FTO → β-catenin m6A demethylation → β-catenin↑ → ERCC1 activity↑ → DNA repair↑	Tumor-intrinsic	ii	FTO promotes resistance (DNA repair via β-catenin)	FTO enhances combined chemo-radiotherapy resistance in CSCC via β-catenin/ERCC1 axis; FTO-β-catenin co-expression predicts poor OS; *in vitro* + *in vivo* + clinical samples	(141)
Diffuse midline glioma (pediatric); patient-derived cultures	Cell cycle dysregulation; FB23–2 inhibitor	Inhibited by FB23-2; global m6A levels elevated in DMG	FTO inhibition → m6A↑ on cell cycle regulators → CDKN1A/GADD45B/TFRC induction → S-phase arrest + replication stress	Tumor-intrinsic	iii	FTO promotes proliferation (cell cycle regulation)	FB23–2 reduces DMG proliferation, survival, induces S-phase arrest; identifies FTO as therapeutic vulnerability in this incurable pediatric cancer; *in vitro* (patient-derived DMG cultures)	(142)
Exosomal and microenvironment-mediated FTO transfer								
NSCLC; gefitinib-resistant patients; serum exosomes	Gefitinib resistance; intercellular exosomal FTO transfer	Enriched in serum exosomes of GR vs GS patients	GR cell exosomal FTO → recipient cells: global m6A↓ → ABCC10 stability↑ (YTHDF2) → drug efflux↑ → acquired gefitinib resistance	Tumor + Intercellular (exosomal)	ii	FTO promotes resistance (exosomal transfer)	Exosomal FTO from GR cells confers gefitinib resistance to recipient NSCLC cells via FTO/YTHDF2/ABCC10 axis; FTO elevated in serum exosomes of clinically resistant patients; *in vitro* + *in vivo*; first description of exosomal FTO-mediated gefitinib resistance	(148)
AML; BM-MSC-derived exosomes to AML cells	Ara-C chemoresistance; BM-niche exosomal transfer	Delivered exosomally from BM-MSCs to AML cells	BM-MSC exosomal FTO → LncRNA GLCC1 m6A demethylation → GLCC1 stability↑ (HuR) → IGF2BP1-c-Myc scaffold → c-Myc activation	Stromal + Tumor (BM-MSC to AML)	ii	FTO promotes resistance (niche-derived exosomal)	BM-MSC-derived exosomal FTO promotes Ara-C chemoresistance in AML via LncRNA GLCC1/IGF2BP1/c-Myc axis; first demonstration of BM-MSC exosomal FTO-mediated AML chemo-resistance; *in vitro* + *in vivo*	(147)
Breast cancer; chemotherapy-treated patients; senescent neutrophil exosomes	Chemotherapy resistance + EMT; senescent neutrophil-mediated	Upregulated in BC tumor cells by exosomal piRNA-17560 (STAT3-dependent)	Senescent neutrophil exosomal piR-17560 → BC: FTO↑ → ZEB1 m6A demethylation (YTHDF2) → ZEB1 stability↑ → chemoresistance + EMT	Myeloid-derived + Tumor (neutrophil exosomal)	ii	FTO promotes resistance (stromal exosomal)	Senescent neutrophil-derived exosomal piRNA-17560 promotes BC chemoresistance and EMT via FTO/ZEB1 axis; piR-17560 serum levels correlate with poor chemotherapy response; *in vitro* + *in vivo* + clinical	(149)

Cellular Compartment indicates the cell type in which FTO is acting: *Tumor-intrinsic*, FTO functions within tumor cells; non-malignant, protective role in healthy tissue (granulosa, cardiomyocyte); Stromal and Myeloid-derived, FTO originating in non-tumor cells delivered to tumor cells via exosomes.

Evidence Tier (per the Evidence Calibration framework in Section 2.1): (i) multi-model *in vivo* with patient-sample correlation; (ii) single *in vivo* perturbation with mechanistic support; (iii) multi-cell-line *in vitro* evidence; (iv) single-cell-line or single-model evidence; (v) correlative bioinformatic evidence only.

Direction & Mechanistic Category indicates whether FTO promotes therapy resistance (oncogenic in the resistance context) or opposes it (sensitizes tumor cells to therapy), followed by the principal mechanistic axis.

**Table 4** exposes three patterns that the original literature-summary format obscured. First, FTO’s directional role in therapy resistance is highly context-dependent: across 32 tumor-context studies (excluding the 3 non-malignant cytoprotective rows), FTO promotes resistance in approximately 24 studies (predominantly upregulated, oncogenic) and opposes resistance in 6 studies (FTO loss promotes resistance, or FTO required for drug cytotoxicity). The remaining 2 studies report drug- or context-dependent direction (e.g., neuroblastoma, bladder cancer). Second, evidence-tier distribution is uneven: Tier ii evidence (single *in vivo* with mechanism) supports 16 studies, while Tier iii (multi-cell-line *in vitro* only) accounts for 14 meaning that approximately 44% of cited mechanistic claims rest on *in vitro* evidence alone, a constraint that should temper inference about generalizability. Third, the three non-malignant cytoprotective findings (cardiomyocyte, granulosa cells) raise a clinically important translational concern: any systemic FTO inhibitor used as a chemosensitizer in tumors may concurrently exacerbate cardiotoxicity or gonadotoxicity in non-malignant tissues, since FTO appears to function as a protective node in those tissues, a consideration absent from current trial-design discussions of FTO-targeted therapy.↑, increased expression, activation, or upregulation; ↓, decreased expression, inhibition, downregulation, or loss; →, mechanistic or regulatory relationship between molecular events.

A well-documented resistance mechanism operates through FTO-mediated suppression of apoptotic mediators. In 5-FU-resistant colorectal cancer, FTO demethylates SIVA1 mRNA to trigger YTHDF2-mediated pro-apoptotic gene degradation (116), while simultaneously stabilizing NUPR1 mRNA to establish an iron-homeostasis buffer via LCN2 and FTH1 that insulates tumor cells from cytotoxic stress (20). Pharmacological FTO inhibition using CS1 in CRC cell lines and patient-derived organoids sensitizes resistant tumors to 5-FU, reverses EMT, impairs stemness, and reduces xenograft tumor formation with clinical relevance reinforced by correlation between FTO overexpression and poor survival in CRC cohorts (117). In breast cancer, STAT3 binds the FTO promoter to drive its expression under doxorubicin pressure, while FTO reciprocally activates STAT3 signaling, creating a self-reinforcing resistance circuit (118). Separately, FTO-mediated stabilization of lncRNA LINC01559 via m^6^;A-YTHDF2-dependent demethylation suppresses miR-1343-3p to enforce docetaxel resistance (119), establishing that a single demethylase can coordinate resistance to mechanistically distinct agents within the same cancer type.

FTO’s resistance program extends into translational control, organellar adaptation, and drug efflux. In gemcitabine-resistant pancreatic cancer, FTO is stabilized through USP7-mediated deubiquitination and demethylates NEDD4 to suppress PTEN and activate PI3K/AKT (120), while independently maintaining LINC01134 stability to license WNT5A/WNT pathway-driven stemness and resistance (121). In gastric cancer, FTO demethylates CDKAL1 to induce mitochondrial fusion, conferring 5-FU resistance through organellar metabolic reprogramming (122). Conversely, omeprazole-mediated FTO suppression activates mTORC1, inhibits pro-survival autophagy, and upregulates the apoptosis-related DDIT3 in an m^6^;A-dependent manner, chemosensitizing gastric cancer cells to multiple agents (123) a pharmacologically exploitable strategy with direct translational relevance. In cisplatin-resistant NSCLC, paradoxical FTO downregulation generates m^6^;A hypermethylation at 5′ UTR regions, excluding eIF3A recruitment and silencing resistance-associated transcripts in an RBM5-dependent manner (124). In bladder cancer, FTO loss similarly promotes resistance to cisplatin, and pharmacological FTO inhibition using MA2 restores cytotoxicity (125). In lung cancer, FTO regulates MRP7 mRNA stability to modulate drug efflux, with FTO inhibition via entacapone restoring paclitaxel sensitivity in resistant cells (126).

Equally important is evidence that FTO can function as a chemosensitizer through activation of alternative cell death programs. In cisplatin-resistant ovarian cancer, FTO overexpression restores sensitivity by activating NLRP3/caspase-1/GSDMD-dependent pyroptosis through NLRP3 demethylation (127), a pro-death function diametrically opposed to its resistance-promoting roles elsewhere. FTO also inhibits doxorubicin-induced ferroptosis in cardiomyocytes via a P53–P21/Nrf2 axis in a HuR-dependent manner (128), cautioning against indiscriminate FTO inhibition in doxorubicin-based regimens. In granulosa cell models, FTO protects against cisplatin cytotoxicity through both Hippo/YAP1 pro-survival signaling (129) and FGF2-driven autophagy activation (130). Neuroblastoma provides a particularly clear illustration of this drug-specificity: FTO sensitizes cells to paclitaxel, has no significant effect on cisplatin response, yet produces opposing effects on etoposide sensitivity depending on expression level (131) arguing against a uniform pro-resistance model.

These findings carry significant limitations. Nearly all mechanistic studies rely on cell lines or xenograft models, without capturing clonal evolution under drug pressure or clinical pharmacokinetic complexity. The temporal dynamics of m^6^;A reprogramming during acquired resistance remain uncharacterized, and attributing resistance to individual downstream targets risks overinterpretation within a multi-pathway system.

Collectively, FTO behaves as a context-dependent epitranscriptomic regulator whose net chemoresistance or chemosensitizing effect is dictated by the functional identity of its m^6^;A-regulated targets in each cancer–drug context. Blanket FTO inhibition will not uniformly sensitize tumors to chemotherapy and may paradoxically promote resistance or organ toxicity where FTO’s pro-death functions are dominant. Systems-level, cancer-type-resolved mapping of the FTO-dependent m^6^;A landscape under specific chemotherapeutic pressures is an essential prerequisite for deploying FTO-targeting strategies with the mechanistic precision required for clinical translation.

### 5.2 Targeted therapy and kinase inhibitor resistance

The capacity of tumors to escape kinase inhibition represents one of the defining challenges of precision oncology, and FTO-mediated epitranscriptomic reprogramming has emerged as a recurrent though mechanistically heterogeneous contributor to this resistance landscape. Across EGFR-targeted therapies and beyond, FTO functions less as a static pro-resistance factor than as a dynamic regulator of signaling plasticity that enables tumor cells to rewire their transcriptional and phenotypic state under therapeutic pressure (**Table 4**).

The EGFR-TKI resistance context provides the most mechanistically dissected illustration. In gefitinib-resistant NSCLC, elevated FTO stabilizes PELI3 mRNA in an m^6^;A-dependent manner, which cooperates with autophagy activation to sustain tumor cell survival against EGFR inhibition; genetic or pharmacological disruption of either PELI3 or autophagy is sufficient to restore gefitinib sensitivity *in vitro* and *in vivo* (132). A parallel but mechanistically distinct resistance axis involves FTO-dependent stabilization of c-Myc, which transcriptionally amplifies the drug efflux transporters BCRP and MRP7, reducing intracellular gefitinib accumulation and maintaining downstream AKT and MAPK signaling (133). Critically, meclofenamic acid a pharmacological FTO inhibitor restores gefitinib sensitivity by dismantling the FTO/c-Myc/efflux transporter axis, demonstrating that epitranscriptomic and pharmacokinetic resistance mechanisms are functionally coupled through a single m^6^;A-regulatory node (133). Together, these recent NSCLC studies (132, 133) reveal that FTO-driven TKI resistance is not mechanistically monolithic: autophagy-mediated survival and transporter-mediated drug exclusion represent parallel, independently sufficient resistance programs that share FTO as their upstream coordinator.

In contrast, loss-of-function FTO contexts generate distinct resistance vulnerabilities that can be therapeutically exploited. FTO downregulation across epithelial cancers promotes EMT through increased m^6^;A modification and altered 3′-end processing of Wnt pathway transcripts, enhancing invasion and metastasis while simultaneously sensitizing tumors to Wnt inhibition (134) a synthetic vulnerability arising directly from the FTO-low, EMT-high phenotypic state. In HIF2α-low clear cell renal cell carcinoma, aberrant FTO activation drives BRD9 mRNA stabilization, enabling SOX17-recruited BRD9 to establish *de novo* super-enhancers that amplify oncogenic programs including CCND1, VEGFR2, and MAPK6; consequently, BRD9 inhibition selectively suppresses FTO-high tumors with greater efficacy than sunitinib in patient-derived xenografts (135). These findings collectively establish that FTO’s resistance function is directional and lineage-specific: its overexpression creates kinase inhibitor resistance in lung cancer while simultaneously generating BRD9 dependency in renal cancer, and its loss creates Wnt inhibitor sensitivity in epithelial cancers.

Critical limitations pervade this body of evidence. The mechanistic studies are overwhelmingly cell-line-based, without prospective clinical validation or pharmacodynamic assessment in patients receiving TKIs. The temporal dynamics of FTO-mediated resistance evolution whether FTO reprogramming precedes, accompanies, or follows kinase inhibitor exposure remain uncharacterized, and clonal heterogeneity within resistant tumors is entirely unaddressed. Furthermore, the field carries a marked EGFR-centric bias, and whether the FTO–signaling plasticity axis generalizes to ALK, RET, or KRAS-targeted therapies is unknown.

Nonetheless, the emerging conceptual model is compelling: FTO functions as an epitranscriptomic regulator of signaling plasticity, enabling tumors to dynamically rewire their dependence on survival pathways under therapeutic pressure. Co-targeting of FTO alongside kinase inhibitors, or exploitation of FTO-status-dependent synthetic lethalities such as BRD9 or Wnt-pathway dependencies, has been proposed as a mechanistically grounded combination strategy. We classify this as a preclinically rationalized hypothesis (readiness level R2): supportive evidence derives from *in vitro* and single-model *in vivo* studies, and the context-specificity of FTO’s resistance polarity has not yet been prospectively characterized in human tumors. Clinical evaluation should therefore be biomarker-stratified from the outset and preceded by independent preclinical reproduction across additional disease-relevant models.

### 5.3 Radiotherapy, temozolomide, and CNS-specific resistance

Resistance to radiotherapy and temozolomide (TMZ) in CNS tumors is not a monolithic phenomenon but a multi-layered adaptive program in which FTO-mediated epitranscriptomic reprogramming plays a convergent coordinating role coupling DNA repair efficiency with stemness maintenance and transcriptional circuit stabilization under therapeutic stress. Integrating evidence across glioblastoma, diffuse midline glioma, and non-CNS tumor models treated with radiation, a unified mechanistic framework emerges in which FTO functions as a master regulator of the resistance niche rather than a modifier of any single pathway (**Table 4**).

At the DNA damage interface, FTO sustains homologous recombination (HR) capacity by maintaining RAD51 focus formation following radiation-induced double-strand breaks. In both HNSCC and glioblastoma stem cells (GSCs), FTO inhibition increases γH2AX persistence and reduces RAD51 availability, impairing HR efficiency and sensitizing cells to ionizing radiation (136, 137). This mechanistic convergence across CNS and non-CNS lineages establishes FTO-dependent RAD51 regulation as a general radioresistance program, though it raises the unresolved question of whether FTO acts directly on HR machinery transcripts or indirectly through broader m^6^;A landscape remodeling.

The stemness dimension is uniquely amplified in the CNS context. GSCs are the principal reservoir of radioresistance in GBM, and FTO inhibition with FB23–2 suppresses GSC self-renewal, reduces tumor sphere formation, and extends survival in intracranial mouse models (137) indicating that FTO supports the stem-like compartment implicated in recurrence. Part of this stemness maintenance operates through FTO-mediated stabilization of VEGFA mRNA; its demethylation sustains VEGFA expression, supporting both vascular programs and autocrine pro-survival signaling within the GSC niche (137). The FTO/m^6^;A/SOX9 axis further reinforces stemness and lineage plasticity in TMZ-resistant GBM: SOX9 is markedly upregulated in resistant cells and is sustained by FTO-mediated demethylation, while pharmacological FTO inhibition via the rhein derivative SYSUP007 downregulates SOX9 and restores TMZ sensitivity (138).

The transcriptional circuit layer introduces additional complexity. In glioma, FTO stabilizes MYC mRNA, enabling MYC to suppress its own antagonist MXI1 through miR-155 and the miR-23a cluster in a self-reinforcing feedback loop; FTO inhibition with MA2 dismantles this circuit and synergizes with TMZ to suppress glioma proliferation (139). Independently, FTO negatively regulates phosducin (PDC), a suppressor of glioma proliferation and TMZ resistance; FTO-mediated PDC repression thus removes a pro-sensitivity factor, and its restoration phenocopies FTO inhibition in enhancing TMZ efficacy (140). In cervical cancer, a non-CNS parallel is provided by FTO-mediated β-catenin mRNA demethylation, which elevates ERCC1 activity and confers chemo-radiotherapy resistance through Wnt-driven DNA repair augmentation (141) a mechanistic principle that may operate in CNS tumors where Wnt signaling is equally active. In diffuse midline glioma, FTO inhibition induces S-phase arrest and upregulates CDKN1A and GADD45B, implicating FTO in cell cycle checkpoint suppression through m^6^;A-dependent regulation of chromosome segregation machinery (142).

Critical limitations constrain interpretation. All studies rely on cell lines or xenograft models without longitudinal patient-derived data capturing resistance evolution under clinical treatment. The spatial architecture of resistant niches and whether FTO activity is enriched within specific GSC subpopulations remains unresolved at single-cell resolution. Crucially, whether FTO is a primary driver establishing resistance programs or a modifier of pre-existing stemness states is mechanistically undetermined across all models, and the integration of radiotherapy and TMZ resistance into a single biological framework rather than parallel independent mechanisms has not been directly demonstrated.

Collectively, FTO emerges as an epitranscriptomic integrator that couples DNA repair capacity with stemness maintenance and oncogenic circuit stability under CNS therapeutic stress. Combining FTO inhibitors with radiotherapy or TMZ prioritizing CNS-penetrant agents such as MA2 or SYSUP007 provides a mechanistically grounded rationale for simultaneously targeting the DNA-repair advantage, the stem-like resistant compartment, and the pro-survival transcriptional programs implicated in GBM recurrence, a hypothesis that requires direct preclinical and clinical testing.

### 5.4 Hematologic resistance and the horizontal-transfer frontier

In hematologic malignancies, FTO has been linked to therapy resistance through two distinct mechanisms: cell-autonomous epitranscriptomic reprogramming within tumor cells, and intercellular transmission of resistance via exosomal cargo. Both axes have been implicated as potential therapeutic vulnerabilities (**Table 4**).

Within tumor cells, FTO drives resistance through convergent stabilization of MYC-centered transcriptional networks across lymphoid and myeloid malignancies. In DLBCL, FTO directly demethylates MYC mRNA to prevent YTHDF2-mediated decay, sustaining MYC protein expression and conferring ibrutinib resistance in a MYC-dependent manner (143). A parallel but mechanistically distinct axis involves FTO-mediated stabilization of FLOT2 mRNA, which activates PI3K/AKT/mTOR signaling and has been associated with DLBCL aggressiveness (144) indicating that FTO can engage multiple oncogenic networks in this disease context. In AML, the resistance program is coupled to a differentiation block: FTO overexpression in relapsed samples hypomethylates FOXO3 mRNA, accelerating its degradation and impairing myeloid differentiation capacity, thereby entrenching the undifferentiated, chemoresistant state (145). Extending beyond hematologic contexts, the FTO/LINK-A/MCM3/HIF-1α axis in esophageal cancer illustrates a mechanistic principle with broad relevance: FTO-stabilized lncRNA LINK-A coordinates cell-cycle progression through MCM3 phosphorylation while simultaneously relieving HIF-1α repression to activate glycolysis and chemoresistance (146). This raises the possibility, not yet established across multiple lineages, that lncRNA-mediated circuit stabilization is a recurring motif of FTO-dependent resistance.

Intercellular (horizontal) transfer introduces an additional regulatory layer of potential therapeutic relevance. Bone marrow mesenchymal stem cell-derived exosomes deliver FTO protein directly into AML cells, where it demethylates lncRNA GLCC1 to stabilize it via HuR binding; GLCC1 then scaffolds the IGF2BP1–c-Myc complex, activating c-Myc-associated survival programs and Ara-C resistance in recipient cells (147). This finding establishes FTO not merely as a tumor cell-intrinsic factor but as a bone marrow niche-derived signal that reprograms AML epitranscriptomes from outside the tumor cell. In NSCLC, gefitinib-resistant cell-derived exosomes deliver FTO to sensitive recipient cells, reducing global m^6^;A levels and upregulating ABCC10 drug efflux transporter via the FTO/YTHDF2/ABCC10 axis directly transmitting acquired resistance to previously sensitive cells both *in vitro* and *in vivo* (148). Senescent neutrophil-derived exosomes complete this intercellular resistance network by delivering piRNA-17560 to breast cancer cells, where it upregulates FTO to destabilize ZEB1 m^6^;A marks, promoting EMT and chemoresistance in a YTHDF2-dependent manner (149). The reported correlation between exosomal piR-17560 levels and poorer chemotherapy response in patients provides preliminary clinical support for this immune-cell-to-tumor resistance axis, although the evidence remains correlative.

Critical limitations require explicit acknowledgment. All horizontal transfer mechanisms are demonstrated primarily *in vitro* or in xenograft systems, without lineage-tracing or quantitative assessment of the contribution of exosomal FTO transfer relative to cell-intrinsic resistance in patient tumors. The temporal dynamics of resistance propagation whether exosomal transfer precedes or follows intrinsic resistance establishment remain uncharacterized, and the spatial distribution of FTO-transferring niche cells within bone marrow or tumor microenvironments is entirely unmapped. The quantitative sufficiency of exosome-delivered FTO protein to meaningfully reprogram recipient cell m^6^;A landscapes has not been rigorously established.

Collectively, these findings outline a working model in which FTO contributes to a distributed resistance network coupling intrinsic epitranscriptomic reprogramming with intercellular resistance transmission. Whether simultaneous disruption of both dimensions, combining intracellular FTO inhibition with strategies targeting exosomal FTO loading or uptake, would yield additive or synergistic clinical benefit remains a hypothesis-generating proposal rather than an experimentally validated strategy. The proposition is conceptually coherent but has not been formally tested in any *in vivo* system, and substantial mechanistic groundwork (defining exosomal FTO functional sufficiency, identifying druggable exosome-loading machinery) is required before clinical evaluation would be appropriate.

## 6 The convergence framework: shared molecular nodes

### 6.1 Rationale and the reader-dependent master model

The reader-dependent organizing logic for FTO function has emerged incrementally from a series of studies over the past several years and is now broadly recognized within the m^6^;A field; it does not, in itself, constitute a novel proposal of this review. What this section attempts is somewhat different: a systematic integration of accumulated evidence that articulates the reader-network logic across cancer contexts and identifies the operational variables that determine FTO output in any given setting. The premise is straightforward: while FTO demethylates m^6^;A marks, the biological consequence of that demethylation is not encoded in FTO itself but is shaped, in part, by which m^6^;A reader proteins engage the newly demethylated transcript in a given cellular context. Under this framing, FTO is better understood not as a driver of cancer per se but as a context-integrating directional switch whose output reflects the reader network, and increasingly, the post-translational reader-state, within which it operates. We treat this framing as a useful synthetic interpretation that organizes a fragmented literature, not as a definitively established mechanism, and we have attempted to enrich it by integrating the PTM-dependent reader regulation, transcript-level reader competition, and substrate-specificity considerations developed in the preceding sections.

The mechanistic logic of this network is often articulated through the YTHDF2–IGF2BP axis, which provides a useful conceptual heuristic rather than a fixed binary rule. YTHDF2 has been most extensively characterized as recruiting the CCR4–NOT deadenylase complex to drive m^6^;A-dependent mRNA decay, while IGF2BP1–3 is best known for shielding m^6^;A-modified transcripts from degradation and stabilizing them. Within this framework, FTO-mediated demethylation removes the shared substrate of both readers, and the net effect on transcript fate depends on which reader was previously dominant. When YTHDF2-mediated decay is the prevailing fate, as with PKM2 in oral cancer (150), FLAD1 in HCC (17), and GPX4 in colorectal cancer (151). FTO demethylation stabilizes these transcripts and produces oncogenic metabolic or immune-evasive outputs. When IGF2BP2-mediated stabilization is the operative reader context, as with HK2 in colorectal cancer (39) and GAS5 in breast cancer (38). FTO loss paradoxically increases m^6^;A methylation, enabling IGF2BP2 to stabilize glycolytic or tumor-suppressive circuits. The GAS5 example is conceptually pivotal: FTO-mediated demethylation of this lncRNA reduces its stability in an IGF2BP2-dependent manner, attenuating a GAS5–IGF2BP2–QKI tumor-suppressive scaffold (38), demonstrating that FTO can suppress anti-tumor non-coding RNA programs through the same enzymatic mechanism that stabilizes oncogenes elsewhere.

However, the YTHDF2–IGF2BP dichotomy should be viewed as a useful heuristic rather than a fixed binary model. Although YTHDF2 is classically linked to CCR4–NOT-dependent decay of m^6^;A-marked transcripts, it can also promote translation in specific tumor contexts; for example, in ovarian cancer, YTHDF2 cooperates with eIF3F and DDX1 to enhance translation of targets such as CKAP2, contributing to paclitaxel resistance (152, 153). Conversely, IGF2BP proteins are best known for stabilizing m^6^;A-modified RNAs and enhancing translation, but they also participate in RNA localization, storage, and ribonucleoprotein organization (154, 155). Reader function is further shaped by post-translational regulation, including YTHDF2 SUMOylation at K571, EGFR/SRC/ERK-dependent stabilization of YTHDF2, and phosphorylation-dependent remodeling of IGF2BP1-containing RNP condensates (156–158). Thus, FTO output cannot be predicted from reader abundance alone, but also depends on transcript identity, competing reader occupancy, cellular signaling state, and reader post-translational status.

The colorectal cancer finding that FTO and ALKBH5 co-operatively restrain HK2 expression via IGF2BP2 (39) while FTO separately stabilizes KCTD15 to activate p53 through HDAC1 suppression (34) illustrates that within a single cancer type, FTO can simultaneously promote tumor-suppressive and oncogenic transcript fates depending on the specific reader–transcript combination. This is not a contradiction but rather the expected outcome of a regulatory node whose output depends on the specific transcript-reader combination engaged.

Critical limitations persist. Quantitative understanding of reader competition, how relative YTHDF2 versus IGF2BP abundance, together with their post-translational activation states, determines transcript fate at individual m^6^;A sites, is largely absent from the current literature. Studies overwhelmingly characterize single FTO–reader–target axes in isolation, without simultaneous profiling of competing reader activities or multi-transcript network dynamics. The temporal evolution of reader composition under oncogenic stress or therapeutic pressure remains unresolved.

These limitations define the conceptual frontier: advancing from FTO-centric, single-target mechanistic models toward reader-network-centric frameworks that treat m^6^;A regulation as an emergent property of competing reader activities across the transcriptome. Systems-level modeling of reader competition, integrated with context-specific transcriptomic and proteomic profiling, will be essential to predict the functional output of FTO activity in any given tumor and to deploy epitranscriptomic therapeutic strategies with the precision that the biology demands.

### 6.2 Metabolic-immune coupling

A useful integrative observation emerges from juxtaposing the metabolic and checkpoint sections above. The pan-tumor glycolytic-immune-evasion axis (93), the FLAD1–PD-L1 axis in HCC (17), and the hypoxia–HIF-1α–FTO–PDK1–PD-L1 cascade in breast cancer (107) together indicate that glycolytic reprogramming and PD-L1 upregulation can be coupled outputs of overlapping FTO-regulated transcript networks, with PDK1 in particular providing a single transcript whose stabilization simultaneously amplifies glycolytic flux and licenses AKT/STAT3-driven PD-L1 transcription (107). This coupling does not appear to be obligatory: the Liu et al. pan-tumor study indicates that glycolytic competition can be functionally sufficient for CD8^+^ T-cell suppression independent of direct PD-L1 regulation (93), suggesting that the two outputs are mechanistically linked but separately tunable across tumor contexts. No published study has, to our knowledge, simultaneously measured glycolytic flux and PD-L1 expression under FTO perturbation in a single experimental system, leaving the relative causal hierarchy unresolved. The translational implication is operational rather than novel: combination strategies pairing FTO inhibition with checkpoint blockade and glycolytic inhibitors warrant evaluation in tumors where both outputs are operative, with reader-network and metabolic profiling potentially identifying patients most likely to benefit.

### 6.3 Ferroptosis and stemness: the resistance-immunity bridge

A notable feature of FTO biology is that its functional consequences extend beyond canonical survival signaling into two cellular programs that have been examined largely independently in the literature: regulated ferroptotic cell death and cancer stem cell (CSC) maintenance. While these programs are mechanistically distinct, they exhibit a conceptual parallel that may warrant integrated investigation: in each, FTO has been reported to attenuate fate-committing signals — pro-ferroptotic transcripts in one case, differentiation-promoting transcripts in the other. Whether this parallel reflects a coordinated, shared regulatory logic operative within the same tumor cell, or rather two independent, lineage-specific FTO functions that happen to share a directional signature, remains an open question that current experimental designs cannot resolve.

The ferroptosis regulatory axis centers on SLC7A11 and GPX4, the two principal effectors of ferroptotic resistance. In colorectal cancer, FTO stabilizes both transcripts through YTHDF2-dependent m^6^;A demethylation, and its suppression whether by genetic knockdown or the novel inhibitor Mupirocin, induces ferroptotic cell death and sensitizes tumors to Erastin and RSL3 (35). In thyroid cancer, the same SLC7A11 axis is operative but directionally inverted: FTO is downregulated and functions as a tumor suppressor by destabilizing SLC7A11 in an m^6^;A-independent manner, triggering ferroptosis to restrain tumor progression (70). This lineage-specific polarity FTO promoting ferroptotic resistance in CRC yet enabling ferroptosis in thyroid cancer recapitulates the reader-dependent master model: the biological outcome is determined by transcript-specific m^6^;A dynamics rather than demethylase activity per se. In glioblastoma, the IDO1–AhR axis transcriptionally represses FTO at the promoter level, reducing m^6^;A methylation on SLC7A11 mRNA and stabilizing it to suppress ferroptosis coupling an immunosuppressive tryptophan catabolism program directly to ferroptotic resistance through FTO as the epitranscriptomic intermediary (159). This finding is conceptually significant: it positions FTO not as an autonomous regulator but as a node through which upstream immunometabolic signals are transduced into ferroptotic fate decisions.

The stemness dimension reveals a parallel architecture. FTO inhibition attenuates leukemia stem cell self-renewal and suppresses LILRB4-mediated immune checkpoint expression, simultaneously collapsing stem-like and immune-evasive programs (97). In pancreatic cancer, FTO overexpression sustains CSC markers and EMT, while its depletion arrests cells in G1, induces MET, and impairs tumor formation establishing FTO as a regulator of the epithelial plasticity continuum (63). In colorectal cancer, cytoplasmic FTO operates through m^6^;Am demethylation rather than canonical m^6^;A, suppressing CSC properties; its loss elevates m^6^;Am levels and enhances *in vivo* tumorigenicity and chemoresistance, while PCIF1 inhibition fully reverses this phenotype (25) expanding FTO’s regulatory scope beyond m^6^;A to a broader cap-proximal epitranscriptomic layer.

Critical limitations persist: ferroptosis and stemness studies are conducted in entirely separate experimental systems, and whether FTO coordinates both programs within the same tumor cell population remains untested. The context-specificity of FTO’s ferroptosis polarity across lineages demands systematic transcriptome-wide analysis before any therapeutic generalization. Targeting FTO to induce ferroptosis while simultaneously depleting stem-like populations represents a mechanistically compelling but experimentally unvalidated combination strategy requiring integrated, lineage-aware preclinical modeling.

## 7 Pharmacological targeting of FTO

### 7.1 Chemical probes and inhibitor development

The pharmacological targeting of FTO has evolved through three conceptually distinct phases, each advancing the field while simultaneously exposing limitations that compound rather than resolve. The foundational phase established structural druggability: rational, binding-pocket-directed design produced the first inhibitors that suppressed FTO m^6^;A demethylase activity, phenocopied genetic depletion in AML by inducing differentiation and apoptosis, and demonstrated *in vivo* efficacy in xenograft systems (44). These probes validated the target but were constrained by suboptimal potency and pharmacokinetic liabilities. The subsequent phase pursued systematic chemical diversification thiophene-based scaffolds achieved improved selectivity for FTO over the closely related eraser ALKBH5, and acylhydrazone derivatives delivered sub-micromolar antiproliferative activity with *in vivo* tumor growth inhibition (160, 161) while structure-guided screening of approved drug libraries identified entacapone as an FTO inhibitor operating through a metabolic FTO–FOXO1 axis (162). The repurposing approach broadened the chemical landscape but introduced pharmacological ambiguity, given entacapone’s established catechol-O-methyltransferase activity and attendant off-target liabilities.

A notable mechanistic development involved a shift in mode of action: targeted FTO degradation elevated m^6^;A methylation on ribosome biogenesis transcripts, routing them to YTHDF2-mediated decay and impairing protein translation with efficacy exceeding catalytic inhibition (163). This established that FTO’s oncogenic functions are not fully reducible to its enzymatic activity, introducing a critical distinction between active-site occupancy and full biological loss-of-function. Across hematologic models, inhibitor-mediated m^6^;A elevation converges on suppression of MYC-driven transcriptional programs, upregulation of RARA and ASB2, and disruption of ribosome biogenesis (160, 164, 165). In solid tumor contexts, FTO inhibition attenuates ERBB3/AKT-mTOR signaling in hepatocellular carcinoma (166) and synergizes with doxorubicin in B-ALL through nucleolar stress and mitochondrial dysfunction (165), demonstrating that inhibitor phenotypes are context-dependent and not uniformly predictable from AML-derived mechanistic models.

Critically, the entire inhibitor landscape remains anchored to hematologic malignancies, with only nascent expansion into solid tumors (166). Selectivity claims against ALKBH5 are routinely made at the enzymatic level but rarely substantiated transcriptome-wide, and no compound has been evaluated within a stratified biological framework accounting for reader composition, tumor lineage, or upstream signaling context. Current FTO inhibitors therefore function as mechanistic probes rather than context-aware therapeutics. Advancing the field requires next-generation compounds integrated into biomarker-defined deployment frameworks pairing inhibitor selection with YTHDF2/IGF2BP2 reader profiling and tumor-type-specific m^6^;A target landscapes to ensure that chemical FTO inhibition is applied where its transcriptomic consequences are both coherent and clinically decisive.

### 7.2 Rational combinations: the triple synthetic vulnerability

The synergy observed when FTO inhibition is combined with mechanistically distinct therapies appears, in several preclinical models, to exceed simple additivity. A plausible explanation is that FTO inhibition concurrently affects multiple oncogenic outputs, epitranscriptomic stability, stress responses, and immune-cell function — increasing tumor vulnerability to partner agents that target related axes. Whether this combinatorial logic generalizes across tumor types remains to be tested.

Along the epitranscriptomic axis, FTO inhibition elevates m^6^;A methylation across oncogenic transcripts, directing them through YTHDF2-mediated decay and collapsing downstream proliferative programs. In breast cancer, this destabilization of MYC and E2F1 mRNAs is substantially amplified by BTK inhibition, whose convergent suppression of the same transcriptional network produces synergy because both agents attack the same oncogenic hub through independent molecular inputs (21). Along the stress amplification axis, FTO loss impairs homologous recombination repair by reducing RAD51 focus formation and compromising homology-directed repair efficiency, rendering cancer cells acutely sensitive to radiation-induced DNA damage in head and neck squamous cell carcinoma (136). Simultaneously, FTO sustains aerobic glycolysis and intratumoral lactate accumulation that suppresses immune cell activation; relieving this metabolic barrier is further compounded by sonodynamic therapy-driven ROS generation and immunogenic cell death, which activate dendritic cell and T lymphocyte infiltration (109). The immune reactivation axis is most directly engaged by anti-PD-1 blockade: FTO suppression increases m^6^;A methylation and YTHDF2-mediated decay of *PDCD1*, *CXCR4*, and *SOX10*, dismantling cell-intrinsic immunosuppressive circuitry and restoring IFNγ sensitivity in an adaptive immunity-dependent manner (23).

Critically, synergy across this framework is not guaranteed, it is axis-dependent and tumor-context-specific. The dominant vulnerability axis varies by lineage: DNA repair deficiency predominates in HPV-negative HNSCC (136), metabolic-immune coupling in melanoma (23, 109), and transcription factor network collapse in breast cancer (21). No systematic comparison across these axes currently exists. Realizing the full potential of this model will require biomarker-guided combination selection matching partner therapies to the dominant FTO-dependent vulnerability axis operative in each tumor context rather than empirical combination screening.

## 8 Critical appraisal: contradictions, caveats, and reproducibility

### 8.1 The contradiction catalogue: same tumor, opposite FTO roles

A recurring and conceptually significant observation in the FTO literature is that, within the same cancer type, FTO has been reported to exert diametrically opposite biological effects. In hepatocellular carcinoma, bladder cancer, prostate cancer, and colorectal cancer, peer-reviewed studies employing comparable methodologies arrive at contradictory conclusions about whether FTO promotes or suppresses tumor progression. Dismissing one set of findings as artifactual is unlikely to constitute an adequate explanation. These contradictions appear robust within their respective experimental contexts, and resolving them represents a substantial conceptual challenge for epitranscriptomic oncology.

**Figure 5:**
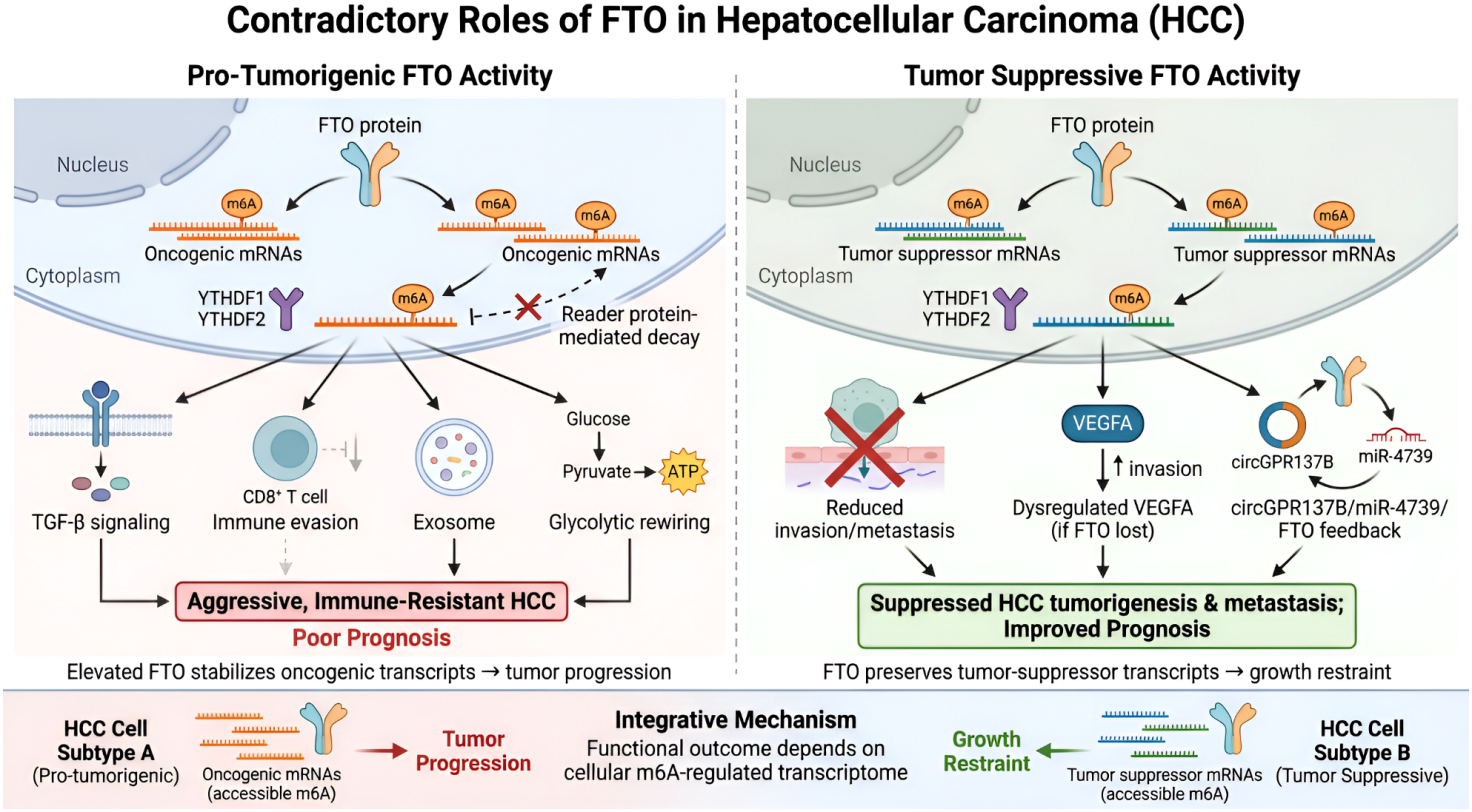
Opposing reported roles of FTO in hepatocellular carcinoma. In oncogenic contexts (left), FTO stabilizes transcripts contributing to TGF-β signaling, metabolic reprogramming, and immune evasion; in tumor-restraining contexts (right), FTO destabilizes pro-invasive transcripts. The directional output appears dictated by the m^6^;A target landscape of distinct HCC subtypes rather than by FTO activity per se.

In HCC, the contradiction is particularly sharp. Several studies associate elevated FTO with poor prognosis and converge on a pro-tumorigenic model in which FTO stabilizes oncogenic programs linked to TGF-β signaling, immune evasion, exosomal suppression of CD8^+^ T cells, and glycolytic rewiring (17, 26, 53, 94). Rather than any single target, the recurring principle is that FTO preserves transcripts that reinforce aggressive, immune-resistant tumor states. Yet an equally rigorous body of HCC evidence positions FTO as a tumor suppressor: its downregulation correlates with metastatic HCC and poor prognosis (27, 28), its loss promotes invasion through dysregulated VEGFA (27), and a circGPR137B/miR-4739/FTO feedback loop establishes FTO as a suppressor of HCC tumorigenesis and metastasis (29). Both bodies of evidence are clinically grounded. The resolution lies less in methodological discrepancy than in the identity of the m^6^;A-regulated transcript network operative in each cellular context: when FTO protects oncogenic RNAs from reader-mediated decay, it promotes progression; when it preserves tumor-suppressive transcripts, the same enzymatic activity becomes growth-restraining. The net biological output depends entirely on which transcripts are most m^6^;A-methylated and reader-accessible in a given HCC subtype (**Figure 5**).

A similar interpretive logic applies in bladder cancer. FTO stabilizes STAT3 mRNA to promote proliferation and migration in one context (30), while in other bladder cancer models FTO is downregulated, its loss promotes proliferation and cisplatin resistance, and its overexpression suppresses invasion by demethylating MALAT1 and NOTCH1 transcripts (31, 32, 125). In prostate cancer, the contradiction is equally stark and better mechanistically resolved: FTO deletion enhances PC-3 cell motility and EMT through IGF2BP2/3-mediated stabilization of DDIT4 (33), while FTO overexpression consistently suppresses PCa proliferation, invasion, and metastasis across multiple studies by stabilizing tumor-suppressive targets including FOXO3a (68), miR-139-5p (67), CLIC4 (69), and EGR2 (36) and FTO is transcriptionally upregulated by ZFHX3 loss to drive tumor-promoting m^6^;A reprogramming (37). Collectively, the prostate cancer data suggest that FTO is predominantly tumor-suppressive in this lineage under baseline conditions, yet can become pro-tumorigenic when upstream alterations, such as ZFHX3 loss, redirect its activity toward a different transcript network.

In colorectal cancer, FTO simultaneously stabilizes both oncogenic mediators NUPR1 (20), ZNF687 (77), SLC7A11/GPX4 (35) and tumor-suppressive ones KCTD15 to activate p53 (34) with net oncogenic dominance in most experimental systems (117). The same YTHDF2-dependent mechanism that produces ferroptotic resistance via SLC7A11 stabilization also produces tumor suppression via KCTD15 stabilization; FTO’s oncogenic or tumor-suppressive identity in CRC is determined by the relative m^6^;A stoichiometry and reader availability across these competing target transcripts.

These contradictions expose a fundamental limitation in the field’s analytical framework: the single-target mechanistic study, however rigorous, captures only a slice of FTO’s regulatory output and cannot predict net biological direction. The literature’s frequent characterization of FTO as uniformly oncogenic may partly reflect publication bias toward high-FTO, aggressively growing tumor contexts rather than the full biological range of FTO function. Quantitative assessment of competing reader abundance—particularly YTHDF2 versus IGF2BP family members—together with transcriptome-wide m^6^;A mapping would help define which transcript networks are functionally dominant in each subtype.

The conceptual advance demanded by this contradiction catalogue is a transition from gene-centric to network-centric models of epitranscriptomic regulation: FTO is neither oncogene nor tumor suppressor but a context-switching regulatory node whose biological polarity is an emergent property of the reader–transcript network it operates within. Therapeutic targeting of FTO should therefore be preceded by systematic characterization of the dominant m^6^;A target landscape in each tumor subtype; otherwise, FTO inhibition may inadvertently disrupt tumor-suppressive demethylation programs in contexts where its net function is restraining, rather than driving, malignant progression.

### 8.2 Methodological limitations and off-target concerns

A candid appraisal of the FTO literature reveals that many published claims, while mechanistically plausible, remain provisional rather than definitive not primarily because the experiments are poorly designed, but because orthogonal validation strategies have been applied inconsistently across the field, limiting the advance from mechanistic association to robust causal inference. Three interrelated methodological limitations combine to produce a body of evidence that is suggestive at the individual study level yet structurally limited when evaluated in aggregate.

The most pervasive weakness is causal insufficiency rooted in incomplete rescue architecture. Across studies spanning cervical cancer, ovarian cancer, and glioma, the dominant experimental design pairs FTO overexpression or knockdown with phenotypic readouts proliferation, apoptosis, invasion and identifies a correlated downstream target. This establishes that FTO perturbation and target expression change together, but does not demonstrate that the identified target is necessary or sufficient for the observed phenotype. In glioma, PDC is proposed as a functional FTO target because PDC overexpression attenuates FTO-driven proliferation, yet whether PDC restoration fully rescues FTO-knockdown phenotypes the bidirectional rescue standard required for pathway necessity is incompletely established (140). Similarly, FGF2 is nominated as the mechanistic effector of FTO in cervical cancer based on co-expression and knockdown correlations without full rescue validation (51). Without symmetric rescue experiments in both gain- and loss-of-function directions, the distinction between a pathway mediator and a co-regulated bystander cannot be established, and the risk of identifying incidental rather than causal targets is substantial.

The second limitation concerns model-system reductionism. The overwhelming majority of FTO studies employ two-dimensional cell line models or immunodeficient xenografts, systems that lack functional immune components, stromal architecture, and intratumoral heterogeneity. Studies reporting FTO effects on autophagy and AKT signaling in ovarian cancer (59) or drug sensitivity in neuroblastoma (131) are conducted in systems that cannot capture the conditional logic reader competition, metabolic state, immune context that the contradiction data across HCC, bladder, and prostate cancer demonstrate to be functionally decisive. When *in vivo* validation is included, it typically confirms cell-line findings rather than interrogating the tumor microenvironmental determinants of FTO function.

The third and most underappreciated limitation involves pharmacologic ambiguity. Meclofenamic acid is deployed as an FTO inhibitor across multiple studies (133), yet its primary pharmacological identity is that of a cyclooxygenase-inhibiting NSAID with well-characterized anti-inflammatory and prostaglandin-suppressing activities that are entirely independent of m^6^;A demethylation. Attributing the sensitization effects of meclofenamic acid to FTO inhibition without demonstrating that an FTO-null background occludes the drug’s activity or that catalytically inactive FTO mutants phenocopy it inflates confidence in FTO-specific interpretations. The same concern applies to omeprazole, whose m^6^;A-elevating effects in gastric cancer (123) are presented as FTO-mediated without excluding proton pump-dependent or FTO-independent transcriptional mechanisms. By contrast, structure-validated inhibitors such as FB23-2, developed through direct FTO binding confirmation and selectivity profiling (44), and entacapone, validated biochemically against purified FTO with substrate-level evidence (162), represent the methodological standard that repurposed agents rarely meet.

The key methodological gap is therefore not a shortage of data but the absence of a validation triad genetic perturbation, biochemical target engagement, and rescue design applied consistently within the same experimental system. Until this triad becomes the field’s methodological baseline, FTO’s causal contributions to specific oncogenic phenotypes will remain mechanistically compelling but epistemically provisional.

### 8.3 Clinical translation gaps and safety considerations

The translation of preclinical FTO-targeting strategies into clinical interventions confronts a fundamental biological problem that the field has not yet adequately addressed: FTO is not a cancer-specific vulnerability but a broadly expressed epitranscriptomic regulator whose activity is required for normal tissue homeostasis across multiple organ systems. Framing FTO inhibition as a straightforward anti-oncogenic strategy may not adequately reflect the complexity of organism-level FTO biology and may convey a degree of therapeutic confidence that current evidence does not support.

The most direct challenge to systemic FTO inhibition is its cardioprotective function. FTO expression in cardiomyocytes is downregulated by doxorubicin, and its restoration activates a P53–P21/Nrf2 ferroptosis-suppressive circuit that preserves cardiac function under chemotherapy-induced oxidative stress (128). This finding reveals that FTO inhibition in a patient receiving doxorubicin-based chemotherapy would simultaneously suppress tumor epitranscriptomic programs and dismantle a cardioprotective mechanism an on-target toxicity that no current FTO inhibitor program has systematically evaluated. The risk is not hypothetical; it is mechanistically defined.

Beyond cardiotoxicity, FTO’s role in non-malignant proliferative disease extends the safety concern. In endometriosis, FTO is overexpressed in ectopic lesions in response to estrogen and inflammatory signals, driving stromal cell invasion via GEF-H1/RhoA in a YTHDF1-dependent manner, and FTO inhibition suppresses lesion growth *in vivo* (167). While therapeutically appealing in isolation, this observation illustrates that FTO inhibitors would exert biologically meaningful effects in hormonally and immunologically active non-malignant tissues—with unpredictable consequences for normal endometrial function, immune regulation, and metabolic homeostasis.

Across the FTO literature, claims of therapeutic promise are built almost exclusively on short-term tumor response endpoints in immunodeficient models, without organism-level safety assessment, tissue-specific pharmacodynamic profiling, or long-term toxicity evaluation. The context-dependence of FTO function tumor-suppressive in prostate and thyroid cancer yet oncogenic in AML and CRC means that a systemic inhibitor could simultaneously suppress one tumor while promoting progression in a second malignancy or protecting critical normal tissues.

What is required is not merely more preclinical data but a conceptual reorientation toward a selective vulnerability model: FTO targeting should be pursued only in tumor contexts where FTO’s net function is demonstrably pro-tumorigenic, where inhibitor delivery can be restricted to the tumor compartment, and where biomarker-guided stratification identifies patients whose normal tissue FTO programs are least likely to be disrupted. Integration of safety endpoints into early-stage mechanistic design is not optional—it is the prerequisite for responsible clinical translation.

### 8.4 Biomarker validation gaps

The proliferation of FTO-related biomarker studies has generated models with high statistical performance in retrospective analyses whose clinical utility remains, upon inspection, largely undemonstrated. The central problem is a systematic conflation of predictive performance measured by AUC values, hazard ratios, and survival stratification with clinical and biological validity, which requires mechanistic anchoring, prospective verification, and independence from confounding tumor-state variables. These are not equivalent, and the field has pursued the former while neglecting the latter.

Pan-cancer analyses correlating FTO expression with immune infiltration, tumor mutational burden, microsatellite instability, and checkpoint-gene expression across cancer-genomic cohorts (90) yield biologically plausible associations but cannot resolve whether FTO is a driver of these immune phenotypes or a surrogate readout of broader tumor-state differences, including tumor purity, stromal composition, and proliferative index, that co-vary with FTO expression without being causally downstream of it. The same limitation applies to machine-learning risk models in colorectal cancer that incorporate FTO alongside seven other m^6^;A regulators (114): when FTO’s independent predictive contribution cannot be isolated from the ensemble signal, and when explanatory analyses (e.g., Shapley additive explanations) identify other regulators as the primary contributors, labeling the composite an “FTO-related biomarker” overstates FTO’s specific role. In acute myeloid leukemia, risk models integrating m^6^;A and immune features (115) demonstrate external validation in retrospective transcriptomic datasets but not prospective clinical validation in patients receiving defined treatment regimens, a critical distinction between a statistical classifier and a clinically actionable tool.

Three structural gaps undermine the translational potential of these models collectively. First, all rely on retrospective bulk transcriptomic datasets whose tumor-purity and immune-composition confounders are computationally estimated rather than experimentally controlled. Second, the gene signatures selected are algorithm-dependent and dataset-sensitive, raising reproducibility concerns that internal cross-validation cannot resolve. Third, and most fundamentally, none of the models establishes that FTO perturbation rather than co-regulated m^6^;A network state causally determines the immune or prognostic phenotype being predicted.

FTO-based biomarkers currently function as integrative readouts of epitranscriptomic tumor state rather than as independent predictive variables. Elevating them to clinical tools requires a fundamental methodological shift: prospective cohort validation, functional dissociation of FTO-specific from m^6^;A-network contributions, and spatially resolved immune profiling that distinguishes FTO-driven immune exclusion from confounded stromal signals.

## 9 A roadmap forward: testable predictions

### 9.1 Near-term clinical translation: reader-stratified combination trials

The convergence of mechanistic and preclinical pharmacologic evidence outlines a plausible, but as yet clinically untested, pathway toward FTO-targeted immunotherapy. We emphasize that no FTO inhibitor or FTO-degrader has, to our knowledge, completed Phase I evaluation in oncology indications, and the framework outlined below should therefore be understood as a preclinical-evidence-based design proposal rather than a near-term clinical roadmap. The translational utility of any FTO-directed strategy is unlikely to be uniform across tumors. Rather, the emerging data support a biomarker-guided model in which the clinical utility of FTO inhibition is likely to depend on molecular context, particularly the composition of m^6^;A reader networks. In melanoma, FTO-mediated m^6^;A erasure preserves protumorigenic and immune-resistant transcripts, including PDCD1, CXCR4, and SOX10, by counteracting reader-dependent RNA decay, thereby reducing sensitivity to anti-PD-1 blockade (23). Parallel work indicates that FTO also reinforces glycolytic reprogramming and lactate accumulation, creating a metabolic barrier to effective T-cell function that can be relieved by FTO inhibition (109). In hematologic settings, FTO further sustains stemness-associated and immune-evasive programs, including checkpoint-related pathways such as LILRB4, which are reversible with pharmacologic inhibition (44, 97). Taken together, these findings argue less for single-agent deployment than for rational combination strategies that integrate FTO inhibition with checkpoint blockade.

A plausible near-term clinical design would therefore test FTO inhibitors in combination with anti-PD-1/PD-L1 agents while prospectively incorporating biomarker stratification. The strongest conceptual candidate is m^6^;A reader composition. Because the biological consequence of FTO inhibition depends on whether methylated transcripts are preferentially routed toward degradation or stabilization, tumors enriched for YTHDF2-like decay programs may be more likely to derive benefit than those dominated by IGF2BP-associated transcript preservation. Early-phase trials could therefore incorporate YTHDF2/IGF2BP2 balance as an exploratory stratification variable rather than an established selection criterion, allowing prospective assessment of whether reader architecture defines a therapeutic window.

Pharmacogenomic markers may provide an additional, though still preliminary, layer of enrichment. Retrospective studies suggest that germline FTO polymorphisms are associated with differential ICI outcomes across solid tumors (112), while other variants appear to influence cancer susceptibility and FTO-linked expression states (88). At present, these data are insufficient for standalone clinical eligibility decisions, but they could be incorporated alongside tumor transcriptomic signatures as exploratory biomarker modules in basket-style trials.

The evidentiary base is still largely preclinical or retrospective, with no prospective validation of reader-based stratification, no clinically standardized assay framework, and unresolved questions regarding dose, toxicity, and tissue-specific dependency. Even so, early-phase FTO inhibitor plus anti-PD-1/PD-L1 trials with mandatory biomarker co-enrollment represent a scientifically justified and clinically testable next step toward context-aware epitranscriptomic immunotherapy.

### 9.2 Mechanistic frontiers: resistance to FTOi and compensatory pathways

Resistance to FTO inhibition appears to arise less from single-pathway escape than from network-level compensation intrinsic to the m^6^;A regulatory system. A proximate mechanism is eraser redundancy: FTO and ALKBH5 co-operatively sustain m^6^;A hypomethylation across overlapping oncogenic loci, such that selective FTO inhibition may be functionally buffered by residual ALKBH5 activity (39). This redundancy is not merely passive. Combined disruption of FTO/ALKBH5 rebalances writer expression, increasing METTL3 while reducing METTL14, thereby restructuring transcript-specific m^6^;A deposition patterns rather than uniformly elevating the methylome and, in some contexts, preserving oncogenic signaling (39). FTO inhibition may therefore reshape the epitranscriptomic landscape rather than simply extinguish it.

Transcript-level rescue further reinforces this architecture. IGF2BP2 can engage hypermethylated targets, including HK2 mRNA, in a stabilizing, m^6^;A-dependent manner, preserving glycolytic flux and FOXO-associated proliferation despite demethylase suppression (39). In this framework, FTO inhibition does not uniformly destabilize oncogenic transcripts; instead, it may shift selective pressure toward reader-mediated rescue, converting hypermethylation into a pro-survival output through altered RNA fate determination.

A parallel challenge emerges at the immune interface. FTO is embedded within a broader regulatory network that includes METTL3, YTHDF1/2, and HNRNP-family proteins, all of which are linked to PD-L1/PD-1 expression and immune infiltration patterns in gastric cancer (98). FTO inhibition alone may therefore be insufficient to fully dismantle checkpoint-supportive circuitry, as residual writers and alternative readers may sustain immune-regulatory transcripts through parallel routes. At the chromosomal level, FTO also regulates centrosome integrity and mitotic fidelity via NuMA and KIFC1 localization (45). While this creates a therapeutic vulnerability in chromosomally unstable tumors, it may also impose selective pressure favoring mitotic stress-tolerant subpopulations with greater adaptive capacity.

Collectively, these observations support a model of epitranscriptomic plasticity–driven resistance, in which the m^6^;A network compensates for single-node inhibition through eraser redundancy, reader-mediated transcript rescue, and pathway-level rewiring. FTO is therefore not an isolated demethylase target but a dynamic node within a compensatory regulatory system. Durable therapeutic responses will likely require systems-level co-targeting combining FTO inhibition with reader blockade or writer modulation to restrict the adaptive escape routes that single-agent FTO inhibition is likely to expose or select for.

### 9.3 Synthetic lethal combinations: the ferroptosis opportunity

Among the multiple regulatory nodes that govern ferroptotic execution, FTO has been identified in several tumor contexts as one m^6^;A-dependent contributor to the post-transcriptional stability of SLC7A11 and GPX4 mRNAs, the two principal effectors of the anti-ferroptotic axis. Through limitation of m^6^;A deposition and consequent attenuation of YTHDF2-mediated decay, FTO can sustain SLC7A11/GPX4 transcript levels and thereby help restrain lipid peroxide accumulation and iron-dependent cell death in tumor types where this circuit is operative. In colorectal cancer, high FTO expression sustains SLC7A11 and GPX4 transcript stability by limiting their m^6^;A methylation and thereby preventing YTHDF2-mediated decay, establishing a ferroptosis-resistant transcriptional state (35). This checkpoint is upstream-regulated by AKT signaling: AKT inhibition transcriptionally downregulates FTO, elevating m^6^;A methylation at a defined GPX4 regulatory site and directing YTHDF2-dependent degradation a ferroptotic signal further amplified when co-occurring protective autophagy is suppressed (151). This positions FTO inhibition combined with system Xc⁻ antagonism or direct GPX4 targeting as a mechanistically coherent synthetic lethal strategy: FTO inhibition primes the anti-ferroptotic transcriptome for collapse, while ferroptosis inducers trigger irreversible lipid peroxidation execution.

Critically, this model is not universal. In papillary thyroid carcinoma, FTO paradoxically functions as a tumor suppressor, downregulating SLC7A11 in an m^6^;A-independent manner and thereby promoting ferroptotic sensitivity (70). This apparent contradiction reflects context-dependent transcript targeting, in which the directionality of FTO’s effect on ferroptosis is determined by tissue-specific m^6^;A target repertoire, reader composition, and site-specific methylation accessibility. The IDO1–AhR–FTO axis in glioblastoma extends this regulatory architecture: tumor-derived immunosuppressive tryptophan catabolism modulates FTO expression via AhR-dependent transcriptional control, with downstream effects on SLC7A11 mRNA stability that reduce ferroptotic vulnerability in this context (159). This finding directly couples immunometabolic signaling to epitranscriptomic ferroptosis control, nominating IDO1 inhibition as a candidate upstream strategy to derepress ferroptotic sensitivity in tumors that depend on this axis.

Together, these findings support a model that may be described as “epitranscriptomic modulation of ferroptotic vulnerability”: the m^6^;A landscape, and FTO’s activity within it, can contribute to the molecular barrier to ferroptotic execution in a tumor-type-specific and signaling-context-dependent manner. Ferroptosis is not a universal consequence of FTO inhibition but a conditional synthetic lethal state gated by lineage identity, upstream kinase activity, and immune-metabolic inputs. Current evidence remains largely confined to *in vitro* models with limited *in vivo* validation and no integration with the tumor immune microenvironment. Realizing this therapeutic opportunity will require biomarker-guided patient stratification incorporating FTO expression, YTHDF2/SLC7A11 co-expression, AKT pathway activity, and IDO1-AhR status to identify tumors in which the synthetic lethal window between FTO inhibition and ferroptosis induction is maximally and durably open.

### 9.4 Technical limitations of epitranscriptomic methods as constraints on FTO inference

A substantive evaluation of FTO biology requires explicit consideration of the methodological limitations that constrain mechanistic inference across the field. The strength of any claim about FTO function, including most of the mechanisms surveyed in this review, depends on assays whose underlying technical performance is itself a subject of ongoing debate. Four limitations are particularly consequential and merit explicit discussion.

#### 9.4.1 MeRIP-seq reproducibility and quantitative limitations

substrate identifications rely on m^6^;A-immunoprecipitation followed by sequencing (MeRIP-seq) or its derivatives (168). Independent benchmarking studies have shown that peak overlap between technical replicates within the same laboratory is frequently below 50%, with inter-laboratory reproducibility lower still (169, 170). Antibody-based enrichment is inherently semi-quantitative and confounded by RNA fragment length, GC content, and local secondary structure. Stoichiometric m^6^;A mapping methods including miCLIP, GLORI, m^6^;A-SAC-seq, and DART-seq, partially address these limitations but remain unevenly adopted, and the m^6^;A-mark stoichiometry at most “FTO target” sites identified in prior work has not been independently validated by an orthogonal quantitative platform (171, 172). Caution is therefore warranted before treating any single MeRIP-seq–derived FTO target list as the definitive substrate set.

#### 9.4.2 m^6^;A antibody specificity

Commercially available anti-m^6^;A antibodies vary substantially in epitope specificity and cross-react with chemically related modifications. The most important cross-reactivity is with m^6^;Am at the mRNA 5′ cap (N6,2′-O-dimethyladenosine), since FTO demethylates both modifications and m^6^;Am sits within the same sequence context that anti-m^6^;A antibodies recognize (13, 41). Cross-reactivity with m¹A has also been reported under specific conditions, and sequence-context binding bias has been documented across multiple antibody clones. The practical consequence is that a substantial fraction of literature signal attributed to “m^6^;A demethylation by FTO” may, in cap-proximal contexts, reflect m^6^;Am demethylation instead, a distinction with direct functional implications for translation initiation and mRNA stability that is not equivalent to internal m^6^;A regulation.

#### 9.4.3 The m^6^;A versus m^6^;Am distinction

FTO demethylates both internal m^6^;A and cap-proximal m^6^;Am, but with different kinetics and substrate preferences that depend on subcellular localization: nuclear FTO acts predominantly on internal m^6^;A, whereas cytoplasmic FTO shows substantial activity toward m^6^;Am (13). These two activities have non-overlapping functional consequences, internal m^6^;A typically regulates transcript stability via reader-mediated decay or stabilization, while m^6^;Am demethylation influences translation efficiency and cap-dependent mRNA fate. The majority of cancer studies cited in this review do not formally distinguish these two activities, and conclusions framed simply as “FTO-mediated m^6^;A demethylation” may therefore conflate mechanistically distinct events. The colorectal cancer cytoplasmic m^6^;Am study (25) is one of the few that explicitly resolves this question; broader application of m^6^;Am-specific mapping (e.g., m^6^;Am-Exo-seq) across cancer contexts is an unmet need.

#### 9.4.4 Defining direct FTO substrates.

Despite the size of the FTO–cancer literature, the criteria required to establish a transcript as a direct FTO substrate, physical interaction with FTO, m^6^;A or m^6^;Am demethylation at a defined site following FTO perturbation, demonstrable dependence of the functional output on that specific site, and exclusion of indirect transcriptional or trans effects, are met for only a small fraction of reported “FTO targets.” Many proposed substrates derive from a combination of (a) altered transcript abundance following FTO knockdown, (b) changes in MeRIP-seq peak intensity at the transcript, and (c) RIP-PCR for FTO–transcript interaction, none of which, individually or together, formally establishes direct catalytic regulation. The recent application of site-specific mutagenesis of m^6^;A consensus motifs in target transcripts (e.g., GAS5 (38), FLAD1 (17)) represents the current methodological gold standard but has been applied to only a subset of published targets. Readers should therefore interpret the FTO substrate landscape as a working hypothesis under active methodological refinement rather than a settled catalog.

#### 9.4.5 Implications for the present review

We have attempted, where possible, to flag claims whose underlying mechanistic evidence rests primarily on MeRIP-seq, on antibody-dependent assays without orthogonal validation, or on m^6^;A/m^6^;Am-undifferentiated approaches. We acknowledge, however, that complete propagation of these technical caveats through every cited mechanism would render the manuscript unreadable; some level of inference from imperfect data is unavoidable in a synthetic review. The technical limitations outlined above should therefore be treated as a baseline constraint on the certainty of all mechanistic claims in this field, including those discussed in the preceding sections, rather than as a critique of any individual study.

### 9.5 Priority research agenda and concluding vision

The context-dependence of FTO biology — that its functional output depends on tumor lineage, transcript context, and m^6^;A reader composition — is at this point a well-established observation in the epitranscriptomics field, and we do not claim it as a novel contribution. What this review attempts is something more modest and, we believe, more useful: an evidence-calibrated, multi-dimensional synthesis that the FTO literature has not yet received. Specifically, we (i) annotate every mechanistic claim with its underlying evidence tier and apply consistent language calibration accordingly; (ii) systematically separate tumor-intrinsic from immune-cell-intrinsic FTO functions across CD8^+^, CD4^+^, and NK lymphoid lineages and across the myeloid compartment; (iii) integrate the post-translational regulation of m^6^;A reader proteins as a previously underdeveloped layer of the reader-dependent logic; (iv) explicitly catalog the technical limitations of epitranscriptomic methods (MeRIP-seq reproducibility, m^6^;A antibody specificity, m^6^;A versus m^6^;Am discrimination, definition of direct FTO substrates) as constraints on mechanistic inference; and (v) translate reader-network biology into a biomarker-stratified clinical trial design logic for FTO inhibitor–ICI combinations. The conceptual building blocks are largely drawn from prior work; the contribution lies in their integration, calibration, and translational orientation. Among the most urgent priorities for advancing the field is improved spatial resolution. Bulk transcriptomics, by conflating spatially segregated compartments, inevitably generates contradictory mechanistic conclusions. Single-cell dissection further demonstrates that FTO governs cancer stem cell maintenance through the Wnt10b/β-catenin axis in ways invisible to population-level analyses (105), underscoring that coherent interpretation of FTO function requires cell-type resolution mapped within its spatial niche.

Equally urgent is the recognition that FTO biology is intrinsically non-cell-autonomous. Resistance to targeted therapy is propagated intercellularly through exosomal FTO transfer: drug-resistant cells export FTO-laden vesicles that reprogram the m^6^;A landscape of naïve recipient cells, transmitting chemoresistance via the FTO/YTHDF2/ABCC10 axis (148). Senescent neutrophils deliver piRNA-17560 that transcriptionally induces FTO in tumor cells, stabilizing ZEB1 and driving EMT and chemoresistance (149), while BM-MSC-derived exosomal FTO amplifies stemness and cytarabine resistance through LncRNA GLCC1–c-Myc activation (147). These findings suggest that FTO inhibition may need to be considered in the context of non-cell-autonomous resistance mechanisms: suppressing FTO in tumor cells while leaving donor compartments, senescent immune cells, stromal mesenchymal populations, intact may be insufficient to prevent resistance re-seeding through intercellular m^6^;A transfer.

Improved functional modeling will be needed to capture these dynamics. Organoid–immune co-cultures that recapitulate FTO-dependent macrophage polarization (111), combined with exosomal m^6^;A profiling and spatial multi-omics, would help establish whether m^6^;A regulation operates predominantly as a cell-intrinsic switch or as a spatially organized, intercellularly propagated property of the tumor microenvironment. Translating these models into clinically informative findings remains an open methodological challenge.

## 10 Conclusion

The evidence synthesized in this review indicates that FTO functions as a context-dependent regulator in cancer rather than a classical oncogene or tumor suppressor. Its divergent roles across tumor types, oncogenic in AML, HCC, and gastric cancer, but tumor-suppressive in prostate, thyroid, and lung adenocarcinoma, are partly explained by the predominant m^6^;A reader proteins engaged and the downstream fate of its target transcripts. When YTHDF2-mediated decay predominates, FTO demethylation tends to promote oncogenic programs, immune evasion, and therapeutic resistance. Conversely, when IGF2BP-dependent stabilization is prevalent, the same enzymatic activity can support tumor-suppressive pathways.

This context-dependent biology has important therapeutic implications. FTO inhibition should not be assumed to be universally beneficial; in certain contexts, it may inadvertently promote tumor progression or impair normal tissue function, such as cardioprotection during doxorubicin treatment or CD8^+^ T-cell fitness. Future therapeutic development would benefit from moving beyond broad inhibitor application toward precision strategies, including patient stratification based on tumor lineage, m^6^;A reader expression profiles, and key target transcript landscapes. Prospective clinical trials will be necessary to validate proposed combination approaches with immune checkpoint inhibitors, ferroptosis inducers, or metabolic modulators. Overall, while mechanistic insights into FTO biology have advanced considerably, translation into clinical benefit remains limited by the lack of robust biomarkers and prospective validation. Progress in this field will require the same context-aware approach that its underlying biology necessitates.
